# Non-orthogonal Configuration Interaction Study on the Effect of Thermal Distortions on the Singlet Fission Process in Photoexcited Pure and B,N-Doped Pentacene Crystals

**DOI:** 10.1021/acs.jpcc.3c02083

**Published:** 2023-08-10

**Authors:** Xavier López, Tjerk P. Straatsma, Aitor Sánchez-Mansilla, Coen de Graaf

**Affiliations:** † Departament de Química Física i Inorgànica, Universitat Rovira i Virgili, Marcel·lí Domingo 1, 43007 Tarragona, Spain; ‡ National Center for Computational Sciences, Oak Ridge National Laboratory, Oak Ridge, Tennessee 37831-6373, United States of America; § Department of Chemistry and Biochemistry, University of Alabama, Tuscaloosa, Alabama 35487-0336, United States of America; ∥ Institució Catalana de Recerca i Estudis Avançats (ICREA). Passeig Lluís Companys 23, 08010 Barcelona, Spain

## Abstract

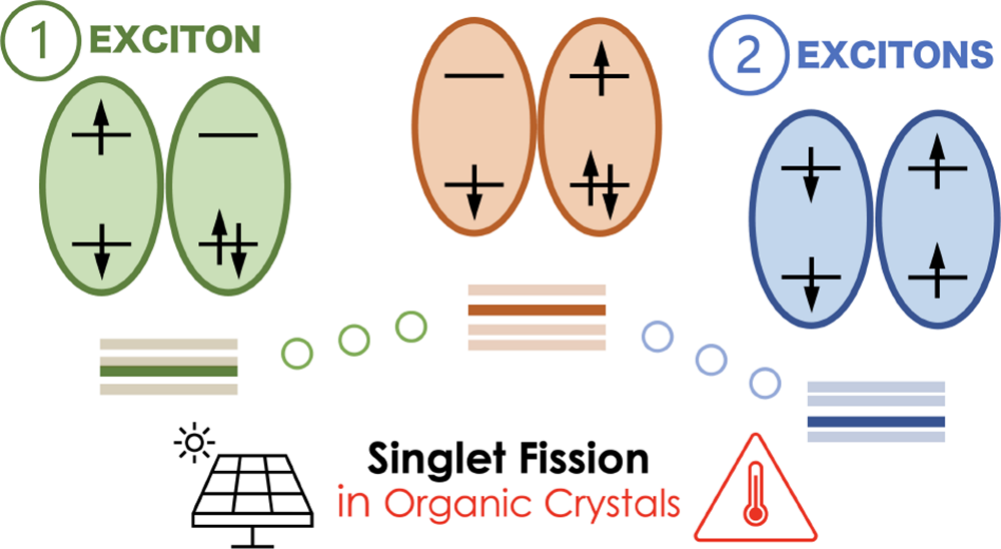

The present computational work analyzes singlet fission (SF) as a pathway for multiplication of photogenerated excitons in crystalline polyacenes. Our study explores the well-known crystalline pentacene (C_22_H_14_) and the prospective and potentially interesting doped B,N-pentacene (BC_20_NH_14_). At the molecular level, the singlet fission process involves a pair of neighboring molecules and is based on the coupling between an excited singlet state (S_1_S_0_) and two singlet-coupled triplets (^1^T_1_T_1_), which, typically, is influenced by an intermolecular charge transfer state. Taking data from periodic density functional theory and ab initio wave function calculations, we applied the non-orthogonal configuration interaction method to determine electronic coupling parameters. The comparison of the results for both equilibrium structures reveal smaller SF coupling for pentacene than for B,N-pentacene by a factor of ∼5. Introduction of the dynamic behavior to the crystals (vibrations, thermal motion) provides a more realistic picture of the effect of the disorder at the molecular level on the SF efficiency. The coupling values associated to out-of-equilibrium structures show that most of the S_1_S_0_/^1^T_1_T_1_ couplings remain virtually constant or slightly increase for pentacene when molecular disorder is introduced. Homologous calculations on B,N-pentacene show a generalized decrease in the coupling values, notably if large phonon displacements are considered. A few of the structures analyzed feature much larger SF coupling if some distortion results in (nearly) degenerate charge transfer and excited singlet and triplet states. For these particular situations, an acceleration of the SF process could occur although in competition with electron–hole separation as an alternative pathway.

## 1 Introduction

The steady increase of global energy demands and the serious rise of the atmospheric CO_2_ concentration triggered the exploitation of alternative and cleaner primary energy sources, such as sunlight, some decades ago. In recent years, the study and development of new and increasingly efficient light-harvesting materials is among the most prominent research interests.^1^ Technology based on silicon heterojunction solar cells has been widely used for capturing sunlight, reaching a record efficiency of 26.8% for a crystalline Si cell (see updated record values for current and voltage relative to the Shockley–Queisser limits in ref (2)),^3^ as recently claimed by the LONGI Green Energy Technology Co., Ltd. (https://www.pv-magazine.com/2022/11/21/longi-claims-worlds-highest-silicon-solar-cell-efficiency). This is not far from the theoretical limit of about 30% for single junction cells.^4,5^ Organic materials are less conventional but promising alternative materials since they can present potential advantages over cells based on mainstream inorganic materials, including a more economical and environmentally friendly production, portability, and flexibility.^6,7^ Therefore, these materials have the potential to be attractive alternatives to the conventional silicon-based cells. However, although the efficiency of solar cells based on organic materials^8^ is steadily increasing, further research and development is needed to design materials with efficiencies similar to, or better than, silicon-based solar cells. In the process of conversion of solar radiation into electricity, low efficiency is the primary limitation for the use of energy from such source. The origin of this limitation is the quantum yield of the process of electronic excitation from the valence to the conduction bands in semiconductors (formation of an electron–hole pair, or exciton) that, ultimately, can generate electrical current. A promising way to significantly increase the efficiency of the excitation process in particular is to use materials that can multiply the initial exciton by a factor larger than one, thereby obtaining more charge carriers than in ordinary processes. This phenomenon has been observed for a multiplying factor of two only, although from a theoretical perspective, it is part of the general multiple exciton generation (MEG) concept,^9^ first observed in anthracene crystals,^10^ and is quite common among the family of acenes, whose general formula for fused benzenes in a linear arrangement is C_4*n*+2_H_2*n*+4_.

**Figure 1:**
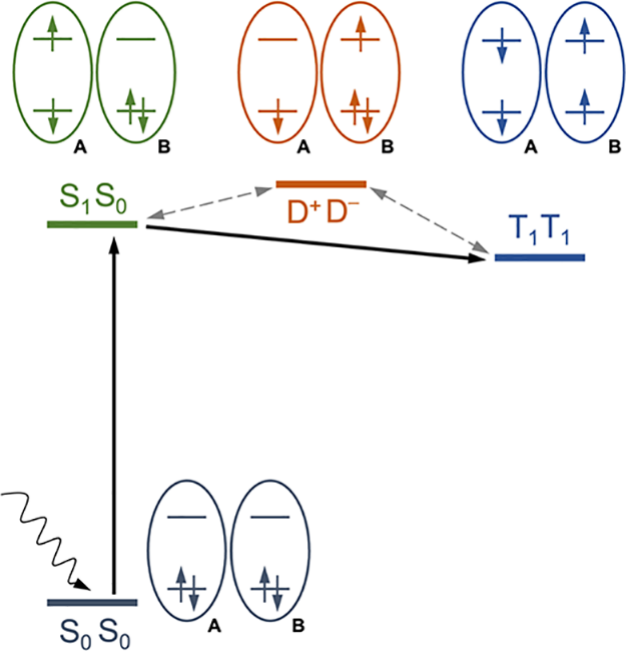
Energy level diagram for the multiexciton generation involving two fragments (A, B). At each step of the process, two orbitals, two electrons, and the state labels are schematically represented for each fragment. S_0_: ground state singlet; S_1_: first excited singlet; T_1_: first excited triplet; D^+^: cationic doublet; D^–^: anionic doublet. See text for details.

Here, we analyze and quantify the singlet fission (SF) process^11,12^ in pentacene and the prospective B,N-substituted pentacene. Although the latter has not yet been reported experimentally, its potential interest is based on similar B,N-substituted acenes,^13−15^ some of which reported to be SF materials. SF can take place between a pair of neighboring molecules AB as schematically depicted in Figure 1. After the initial photoexcitation of molecule A to a higher-lying singlet state represented by A**, the process A** + B → A* + B* takes place, where the excited singlet (A**≡S_1_) on molecule A decays to a lower lying triplet state (A*≡T_1_), accompanied by the generation of an equivalent T_1_ state on molecule B. The overall process can be labeled (S_1_S_0_) → ^1^(T_1_T_1_), where it is indicated that the two final triplets on A and B are coupled to a singlet. In this way, the absorption of one photon generates two excitons.

In the most general way, two conditions must be fulfilled for SF to be effective: (i) the energy of the ^1^(T_1_T_1_) state should be lower than the energy of the initial excited (S_1_S_0_) singlet state, and (ii) their mutual electronic coupling should be significant.^16^ Energy losses and exciton decay via different pathways are factors that can decrease the efficiency of the process and need to be taken into account.^17^ It has been reported that the total coupling is enhanced by a charge-separated state, formally a combination of D^+^D^–^ and D^–^D^+^ configurations, for the pair of molecules involved,^18−23^ as shown in Figure 1. This configuration is not an intermediate state, but a representation of a relevant electron distribution that contributes to both the S_1_S_0_ and T_1_T_1_ states and increases their mutual coupling. The electronic coupling between initial (1) and final (2) states can be calculated from eq 1
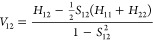
1where *H_ij_* are Hamiltonian interaction elements and *S*_12_ is the overlap integral between states 1 and 2. This equation is usually solved through some simplifications such that the coupling can be estimated from orbital energies and overlaps,^20,24,25^ or from transition dipole moments of the local excited states,^26,27^ among other approximate methods.^28^ Instead of simplifying the calculation of *V*_12_, we explicitly calculate it with the non-orthogonal configuration interaction scheme,^29^ also known as the NOCI-Fragments approach. This computational procedure starts with the generation of a set of monomer (or fragment) wave functions that include full orbital relaxation, static and dynamic electron correlation. These monomer wave functions are then used to construct spin-adapted diabatic states of the whole system, the so-called multielectron basis functions (MEBFs) spanning the NOCI space. NOCI calculations between these MEBFs provide the energies and wave functions of the relevant electronic states, together with the electronic coupling between the diabatic states. In this way, the final wave function expansion remains short yet accurate, facilitating the interpretation of the physics of the system. This facility for interpretation, combined with the increased computer power, is one of the main reasons why NOCI has regained attention over the last decade and various slightly different implementations and applications have been recently reported.^30−38^ For the NOCI calculations, we applied the massively parallel and GPU-accelerated GronOR program,^39^ which provides coupling parameters obtained from the pair states with and without dynamical electron correlation corrections.^40^ However, only the results with dynamical electron correlation corrections are herein reported.

**Figure 2:**
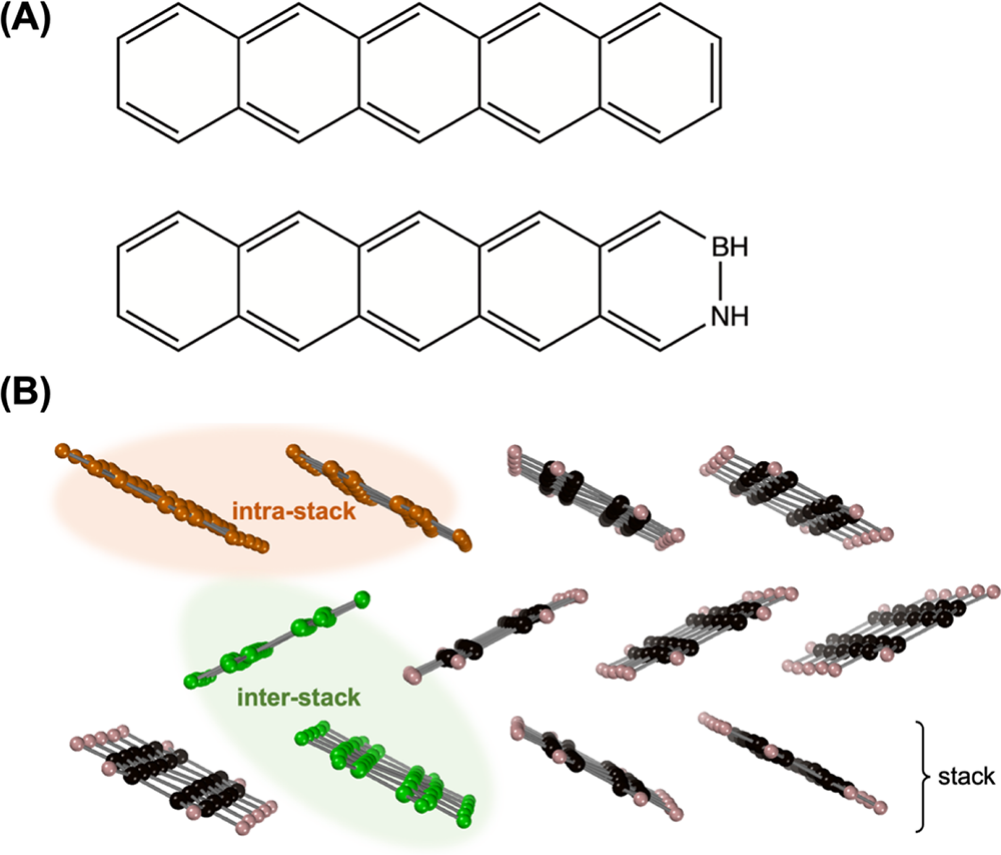
(A) Molecular structure of pentacene (top) and 2,3-B,N-pentacene (bottom). (B) View of three stacks in the crystal of pentacene. Green crosswise molecules represent an interstack pair (pair1) and parallel orange molecules represent an intrastack pair (pair2).

The present work tackles the above questions by focusing on two systems: the well-known pentacene (C_22_H_14_) crystal and a partially doped form of it, which contains B,N-substituted pentacene molecules (BC_20_NH_14_). The latter has not been reported experimentally to date. Their molecular structures are depicted in Figure 2A. Other azaborine derivatives of the acene family, such as pyrene,^13^ perylene,^14^ and tetrabenzopentacene,^15^ have been previously analyzed with promising results regarding the SF phenomenon.

Figure 2B shows the crystal packing and the two types of neighboring monomer pairs (interstack, pair1, and intrastack, pair2) taken from the crystal, for which electronic coupling parameters are calculated. We analyze the effect that vibrational/thermal distortions taking place in the crystal have on the SF process. The study has two different approaches. In one, we analyze the effect of low-energy crystal vibrations (phonons) by applying atomic displacements for each phonon independently, which has been recently experimentally explored for pentacene^41^ and computationally analyzed for tetracene.^42^ In the other, we consider distorted geometries generated with a molecular dynamics simulation. With this approach, we obtain out-of-equilibrium atomic positions in a dynamical way to account for the thermal disorder of the structures. These results are used to assess the extent of the changes in SF coupling, (S_1_S_0_) → ^1^(T_1_T_1_), produced by internal structural changes originating from the thermal disorder in the crystal.

## 2 Methods and Results

### 2.1 Preliminary Calculations on the B,N-Pentacene Structure

Our interest in B,N-pentacene as a prospective material is based on other systems reported, some of them thoroughly analyzed at the computational level. Certain studies include numerous positional isomers of B,N-tetracene,^43−45^ the azaborine polyacene closest to B,N-pentacene. Among the numerous positional isomers of B,N-pentacene, following previously reported studies on the similar B,N-tetracene system,^45^ the objective of this study was to choose one case showing not too large a gap between the highest occupied and the lowest unoccupied molecular orbitals (HOMO-LUMO gap), indicative of chemical stability. The second criterion relates to the fact that *E*(S_1_S_0_) ≥ *E*(T_1_T_1_) for singlet fission to be effective, even more if the process is exoergic. In previous computational work carried out on several isomers of B,N-tetracene, it was concluded that the arrangements with neighboring B-N atoms are optimal for an efficient SF.^44,45^ For B,N-tetracene, it was demonstrated that the 2,3 isomer fulfills requirements of stability and effective SF, together with three other systems. To confirm that this holds for B,N-substituted pentacene, we compared complete active space self-consistent field (CASSCF) and complete active space perturbation theory to second-order (CASPT2) results on the positional isomers 1,2 and 2,3. Isomer 1,2 has *E*(S_1_S_0_) < *E*(T_1_T_1_) by a large difference, not fulfilling the fundamental requirement for SF, whereas *E*(S_1_S_0_) > *E*(T_1_T_1_) for isomer 2,3. See Figure S1 in the Supporting Information (SI) for related numerical details. These facts prompted us to investigate the structure depicted in Figure 2A as an interesting prospective B,N-doped system rather than other positional isomers.

### 2.2 Periodic DFT Calculations

#### 2.2.1 Crystal Composition and Optimization

Here, we present crystal structures obtained from periodic density functional theory (DFT) geometry optimization calculations performed with the VASP 5.3.5 program.^46−49^ The packing of pentacene crystals is of the triclinic system (*P*1̅), with a unit cell containing 72 atoms (two pentacene units) featuring crystallographic parameters^50^*a* = 7.90 Å, *b* = 6.06 Å, *c* = 16.01 Å, α = 101.90°, β = 112.60°, γ = 85.80° and volume cell of 692.38 Å^3^. For the optimization runs, we used a Γ-centered 2 × 2 × 2 *k*-point mesh for both the pentacene and the B,N-pentacene crystals and included as structural degrees of freedom the atomic positions and all six cell parameters as well as the cell volume. The cutoff energy for the plane-wave basis was set to 700 eV, and the convergence thresholds for the self-consistent-field electronic energy and the structural relaxation were set to 10^–7^ eV. We also tested several functionals based on the Perdew–Burke–Ernzerhof (PBE^51^) one to get the optimized coordinates, namely, PBE, RPBE,^52^ RPBE-vdW, and PBE-vdW. The van der Waals (vdW) corrections correspond to Grimme’s DFT-D3 implementation.^53^ When compared to the crystallographic data, the results obtained with PBE and RPBE were less accurate than those with RPBE-vdW and PBE-vdW, the latter functional showing the best match. The C–C bond distances in the optimized crystal lie in the 1.37–1.46 Å range, very close to the X-ray values (1.35–1.48 Å), with an angle of 51.6° between interstack monomers (pair1, see Figure 2B) and shortest interstack C–C distances in the range 3.78–3.91 Å, matching the crystallographic ones (3.72–4.01 Å). The list of optimized atomic coordinates for the pentacene crystal is shown in Table S1 in the SI.

To calculate the doped B,N-pentacene crystal, we generated a model with evenly distributed 50% undoped and 50% B,N-doped molecules. Since no X-ray data is available to compare with, and given the good agreement with experiment obtained with the PBE-vdW functional for pentacene, we rely on the computed optimized structure with this functional. The resulting B,N-doped crystal parameters are very similar to those of the pentacene crystal, as shown in Table 1, with computed B–C, N–C and B–N distances of 1.478, 1.334, and 1.470 Å, respectively, and an angle of 49.9° between non-parallel monomers. The list of optimized atomic coordinates for the 50% doped B,N-pentacene crystal is shown in Table S2 in the SI.

**Table 1:** Structural Parameters for the Pentacene Crystal Obtained by Optimization with Different Functionals with the VASP Program^b^

	*a* (Å)	*b* (Å)	*c* (Å)	α (°)	β (°)	γ (°)	volume (Å^3^)
RPBE	9.62	6.63	16.24	100.11	109.98	87.05	957.5
RPBE-vdW	7.67	5.99	15.83	102.75	113.01	85.26	652.8
PBE	8.88	6.24	15.98	101.78	110.88	86.26	809.4
PBE-vdW	7.80	6.01	15.77	102.44	112.33	85.53	667.2
*PBE-vdW	7.77	6.01	15.97	101.52	113.55	85.43	668.9
X-ray^a^	7.90	6.06	16.01	101.90	112.60	85.80	692.38

a Data from ref (50).

b The parameters for the optimized B,N-pentacene are marked with *.

To guarantee the suitability of the structures utilized in this work and validate the method used, we re-optimized the pentacene and the B,N-pentacene crystals with a 4 × 4 × 4 *k*-point mesh and the PBE-vdW functional. The resulting structures were found to be indistinguishable to the ones with a 2 × 2 × 2 *k*-point mesh, judging by the very similar cell parameters and energies obtained (see Table S3 in the SI). The results above also show the relevance of the van der Waals corrections in the accuracy of the optimized parameters. Therefore, the remainder of the structures discussed and all the results derived are based on the PBE-vdW functional.

#### 2.2.2 Crystal Phonons

Taking the equilibrium structures, we calculated the crystal vibrations for both compounds applying the computational details described above. For the unit cell of 72 atoms (two molecular units), the calculation generates 216 normal modes of vibration, of which the lowest three in frequency are the so-called acoustic modes with frequencies close to zero. The next 27 normal modes correspond to the crystal phonons (*P*1̅ space group), which in the case of pentacene take values in the range 34.4 to 267.4 cm^–1^, and for B,N-pentacene between 33.2 and 267.2 cm^–1^. See Tables S4 and S5 in the SI for the complete list of vibrational frequencies. It stands out that these frequencies do not differ much between the two compounds, as expected, because there is little internal molecular component in these vibrations. We selected the following phonons:
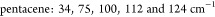

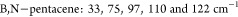
which are of a different nature and cover the basic intermolecular movements, such as the relative bending of the molecular planes, sliding of the units along and perpendicular to the stack direction, etc., see Table S6 and Figure S2 in the SI. The corresponding displacement matrices generated by VASP (full list in Table S7 in the SI) were used to get distorted geometries along the potential energy surfaces of the different phonon movements. We applied multiplication factors of 0.2 and 0.5 to the displacement matrices of all phonons, generating new geometries. From these geometries, we obtained wave functions and coupling parameters. The energy increase per cell produced by the mentioned vibrational distortions for pentacene and BN-pentacene systems is between 0.1 and 1 eV, depending on the displacement factor applied. These increases in energy, as will be shown later, are clearly smaller than the range of energy fluctuations obtained from AIMD simulations at 300 K. We infer that the vibrational motions explored for SF calculations lie within the range achievable by the system at room temperature.

The vibrational calculations also provide information on the stability of the optimized crystals. In addition to the parameters described above, the all-positive (with three near-zero) frequencies and the elastic tensor analysis^54^ (see Table S8 in the SI) confirm that the herein optimized pentacene and B,N-pentacene crystals have both dynamical and elastic stability.

#### 2.2.3 Ab Initio Molecular Dynamics

We carried out ab initio molecular dynamics (AIMD) calculations with the PBE-vdW functional, independently for pentacene and B,N-pentacene doped crystals, with the goal of including the thermal disorder in the systems and extract a series of additional atomic arrangements (*snapshots*) from the calculated trajectory to calculate the SF coupling parameters.

**Figure 3:**
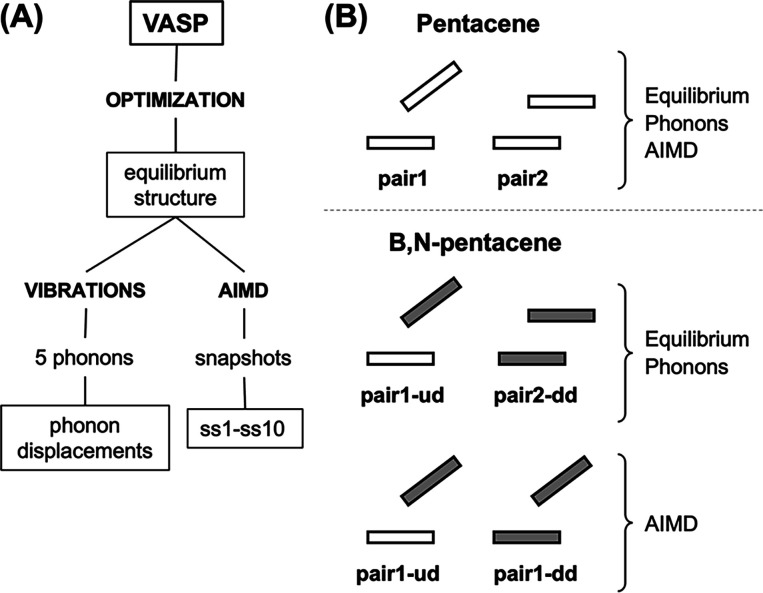
(A) Summary of the VASP runs performed (in capital letters) and the structures obtained (in boxes). (B) Pair geometries used for the study of pentacene and B,N-pentacene crystals. Empty and filled blocks represent pentacene (u: undoped) and B,N-pentacene (d: doped) monomers, respectively. For the B,N-pentacene-containing crystal, the fragment pairs studied and dismissed depend on the calculation type, see text and Figure S3 in the SI for details.

The simulation boxes for AIMD runs are substantially larger than the crystallographic unit cell of 72 atoms to avoid periodicity repeats of the atomic motion at too short a range. The supercells are 3 × 3 × 2 for the pentacene crystal, containing 1296 atoms (36 molecules), and 2 × 3 × 2 for the B,N-pentacene crystal, containing 864 atoms (24 molecules). The use of a smaller box in the latter case is necessitated by the larger memory requirements of the VASP calculation, which arises from the replacement of C by B or N. The 3 × 3 × 2 simulation box of pentacene was generated directly from the optimized crystal. However, for the B,N-pentacene doped case, we introduced two additional B,N-pentacene units to the optimized 50% doped crystal, randomly located. The goal of this manipulation is to introduce and explore an additional coupling mode, namely, the intrastack neighboring interaction between two B,N-pentacene molecules (pair2-dd), not present in the optimized crystal with 50% doping. A summary of the calculations performed with the VASP program is given in Figure 3A.

For the AIMD runs, we applied a temperature of 300 K to a simulation time of 1 ps, with 1000 time steps of 1 fs each. In these calculations, the cutoff energy was set to 400 eV. We imposed an NVT ensemble through use of the Nosé–Hoover thermostat.^55^ The temperature parameter fluctuates around the reference temperature value. The resulting time evolution of temperature and energy for pentacene and B,N-pentacene crystals is shown in Figure S4 in the SI. From each of the two AIMD runs, we extracted 10 fragment pair geometries (snapshots labeled ss1 to ss10), one every 100 fs. The largest energy fluctuations met at 300 K can, in some cases, reach to 2.5–3 eV with respect to the energy at equilibrium and have been taken as reference to assess the length of the phonon-based geometrical displacements. These structures were used to obtain the ab initio wave functions and the NOCI parameters for different pairs of molecules (intra- and interstack; (un)doped-(un)doped).

### 2.3 Fragment CASSCF Calculations

MEBFs for pairs of molecules (AB) were obtained using OpenMolcas,^56^ as the input for GronOR to run NOCI calculations. All CASSCF calculations are based on the atomic basis sets taken from the ANO-RCC library (3s, 1p functions for H; 4s, 3p, 1d functions for C, N and B) and the Cholesky decomposition to approximate the two-electron integrals. The structures of pentacene and B,N-pentacene are those obtained from periodic VASP runs (optimization, vibrational or molecular dynamics) on the corresponding crystals. A summary of the monomer pairs analyzed in this work are shown in Figure 3B and described in detail below.

#### 2.3.1 Pentacene

We considered both monomer pairs, shown in Figures 2B and 3B (top), in equilibrium, phonon, and AIMD-generated geometries. They differ in their mutual orientation, namely, crosswise (inter-stack, pair1) or parallel (intra-stack, pair2), with different couplings between electronic states.

#### 2.3.2 B,N-Pentacene

The structure of the 50% B,N-doped crystal generates more pair interaction types than pure pentacene (see Figure S3 in the SI). From these, the analysis of neighboring pentacene-pentacene (undoped-undoped, uu) interactions were dismissed assuming that they are comparable to those obtained from pure pentacene. Therefore, for equilibrium and distorted phonon structures, we obtained wave functions for pair1 with undoped-doped monomers (pair1-ud) and for pair2 with doped-doped monomers (pair2-dd). For the structures generated during the AIMD run, with extra doped monomers, we analyzed two crosswise pair interactions, namely, the doped-doped (pair1-dd) and undoped-doped (pair1-ud) pairs. As will be shown in the NOCI results section, we dismissed the analysis of the parallel pair2-dd interaction due to its limited impact on SF.

**Figure 4:**
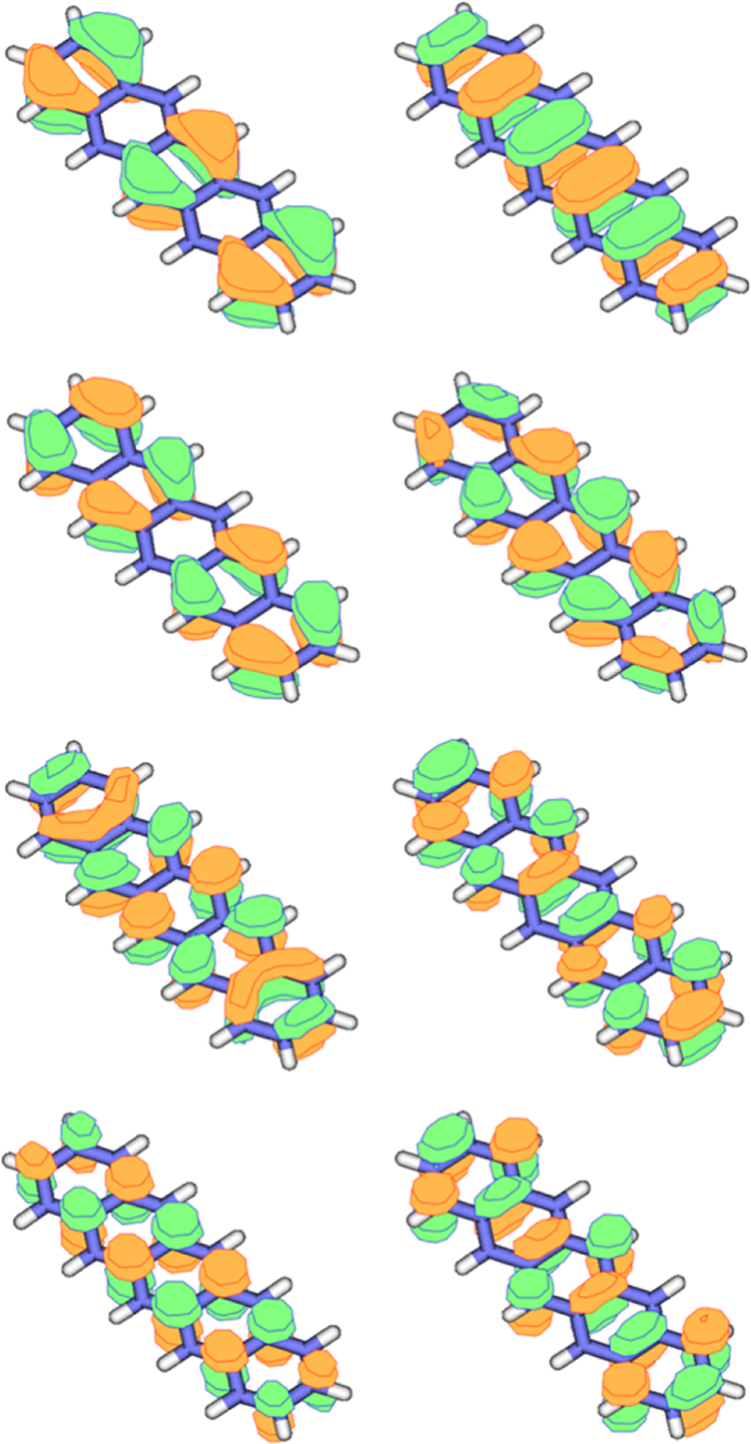
Orbitals of π-like nature constituting the CAS(8,8) for the pentacene monomer.

Complete active space self-consistent field (CASSCF) wave functions were constructed independently for each of these states to ensure a full treatment of the orbital relaxation and the non-dynamic electron correlation. The NOCI-F study of the SF process requires handling several electronic states for each monomer: the ground state (S_0_), the first excited singlet state (S_1_), the lowest triplet (T_1_), the cationic doublet (D^+^), and the anionic doublet (D^–^). The latter two states were used to construct the diabatic representation of the charge-transfer states, in which one electron is transferred from one molecule to a neighboring one. The corresponding active spaces, all containing π-type orbitals, are composed of 8 electrons and 8 orbitals (8,8) for the singlet and triplet states of the neutral molecules (see Figure 4), one electron less for the cationic form (7,8), and one electron more for the anionic form (9,8). The homologous CAS(8,8) orbitals for B,N-pentacene are shown in Figure S5 in the SI.

To check if the active size can affect the conclusions of the work, larger CAS(10,10), (12,12), and (14,14) were tested for the isolated pentacene molecule in its equilibrium gas phase geometry. The results listed in Table S9 (SI) show that a CAS(8,8) is a good choice, mainly given the superior match with experiments^57^ within the set of calculations done as well as the modest variations of the numerical data as the number of orbitals and electrons included in the CAS increases. In addition, the use of a larger active space may have severely limited the number of structures analyzed due to the larger computational cost and, consequently, the generality of the results of this study. All the CASSCF calculations were performed with the geometry of the S_0_ state. The CAS calculations generated 2485 determinants for the singlet states, 3136 for the excited triplet, and 3920 for the ground state doublet of both ionic states.

Taking the CASSCF wave functions for the monomer states, we included the dynamic electron correlation effects by means of perturbation theory to second order, thus obtaining the corresponding CASPT2 energies.^58^ The CASPT2 energy values were obtained with no IPEA correction parameter (i.e., IPEA = 0). Despite not being the present standard in OpenMolcas, this option provides better excited state energies than IPEA = 0.25. See Table 2 for CASSCF and CASPT2 energies for undoped and doped monomers in their equilibrium geometries.

**Table 2:** CASSCF and CASPT2 State Energies^a^ for Undoped (u) and Doped (d) Monomers in Their Corresponding Equilibrium Crystal Geometries

crystal		fragm.	S_1_	T_1_	D^+^	D^–^
pure pentacene	CASSCF	A	3.250	1.504	5.707	0.231
		B	3.259	1.514	5.706	0.242
	CASPT2	A	2.079	0.557	6.153	–1.186
		B	2.080	0.564	6.150	–1.177
50% BN-pentacene	CASSCF	A (u)	3.240	1.494	5.705	0.214
		B (d)	1.803	0.851	5.113	–0.170
	CASPT2	A (u)	2.080	0.553	6.153	–1.143
		B (d)	1.601	0.376	6.109	–1.263

a Relative to the corresponding S_0_ state, in eV units.

Using the CASSCF monomer wave functions, six MEBFs were generated for each pair of monomers: S_0_S_0_, S_1_S_0_, S_0_S_1_, T_1_T_1_, D^+^D^–^, and D^–^D^+^ spanning a 6 × 6 NOCI Hamiltonian. The molecular orbitals that constitute these MEBFs are (i) fully relaxed for each monomer state and (ii) constitute a non-orthogonal set of functions. The use of medium-size active spaces in the present work avoids excessive computational costs derived from the subsequent NOCI calculations without loss of quality of the results.

### 2.4 Dimer NOCI Calculations

The NOCI electronic couplings generated in this study are organized in three parts, depending on the origin of the pair geometries: equilibrium geometry, phonon potential energy surfaces, or snapshots from AIMD runs. Based on the assumption that interfragment dynamical electron correlation is small and that it only has a significant effect on the relative energies but not on the relative importance of the leading configurations in the wave function expansion, dynamical correlation can be accurately accounted for in the NOCI calculations by shifting the diagonal matrix elements with the dynamic electron correlation correction calculated for the monomer electronic states that constitute the MEBF under consideration.^58^

#### 2.4.1 Singlet Fission in Equilibrium Geometries

**Figure 5:**
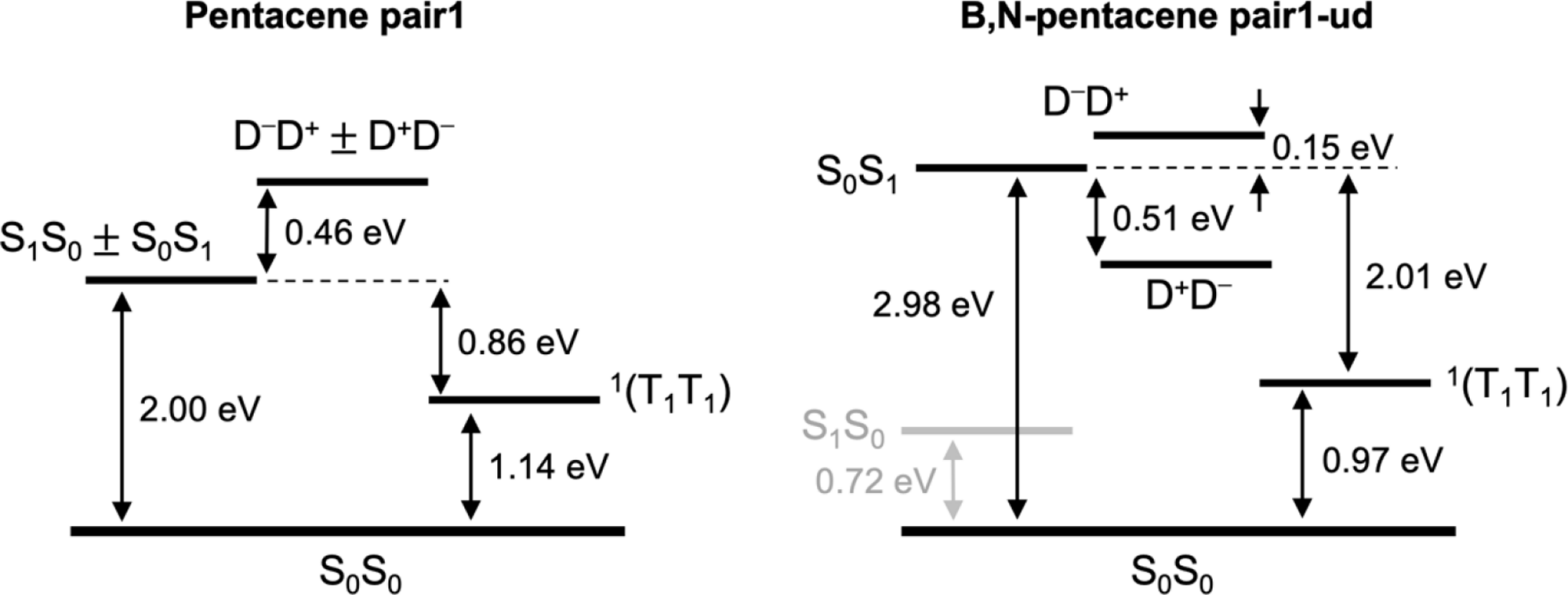
CASPT2 pair state energy gaps for the oblique pairs of pentacene (pair1) and B,N-doped (pair1-ud) crystals. Notable variations appear as the doping moiety is introduced.

Electronic couplings for the S_1_S_0_ → ^1^(T_1_T_1_) process are calculated for pair1 and pair2 dimers for pentacene and for pair1-ud and pair2-dd for B,N-pentacene. Electronic couplings can be obtained such that the initially excited S_1_S_0_ and final ^1^(T_1_T_1_) states are the only ones involved in the process, so in a direct fashion. However, evidence exists that SF is enhanced by D^+^D^–^ and D^–^D^+^ charge-transfer states,^18–23^ as shown in Figure 1. This enhancement of the coupling is included by constructing two new MEBFs as
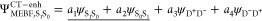

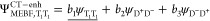
where the underlined terms are dominant in each MEBF (see further discussion in the SI section ‘Charge-transfer enhanced coupling’). The coefficients *a_i_* and *b_i_* are the result of the diagonalization of a 4 × 4 and 3 × 3 subblock of the full NOCI Hamiltonian. The coupling of these MEBFs, calculated by eq 1, reflects the coupling of a local excited singlet state with the double triplet state, enhanced by the ionic configurations. It covers all three mechanisms described for the formation of the ^1^T_1_T_1_ state:^12^ direct formation, the mechanism involving a charge transfer (CT)-enhanced coupling and the two-step mechanism in which the CT states act as the intermediate. Note that the distinction between the CT-enhanced and the two-step mechanism disappears when the CT states become comparable in energy with the S_1_S_0_ and ^1^T_1_T_1_ states. The comparison of the direct and total couplings in Table 3 confirms that the latter are much stronger, reinforcing the assumption that ionic configurations D^+^D^–^ and D^–^D^+^ play a key role in the generation of the multiexciton ^1^T_1_T_1_ state. In Figure 5, with energies obtained from the optimized crystal geometry, the ionic configurations are found 0.46 eV above the singlet excited state, indicating that the CT-enhanced mechanism for ^1^T_1_T_1_ formation is dominant. Values for pair1 dimers of pentacene are much larger than pair2 (parallel orientation) ones, the latter featuring practically zero coupling.

**Table 3:** Direct and Total S_1_S_0_/^1^(T_1_T_1_) Couplings^a^ for Pentacene and B,N-Pentacene in Their Crystal Equilibrium Geometries

crystal	interaction type	direct	total
pentacene	pair1	1.76	4.32
	pair2	0.025	0.500
B,N-doped	pair1-ud	2.58	32.1
	pair2-dd	0.411	1.16

a Absolute values, in meV units. All the values include the dynamical correlation energy corrections at the CASPT2 level.

Finally, the B,N-doped crystal presents electron coupling values substantially larger than those of pentacene, as shown in the bottom row of Table 3. These results indicate that the non-parallel orientation of neighboring monomers dominates the electronic coupling between the S_0_S_1_ and ^1^T_1_T_1_ in the SF pathway, and an increasing factor of 3–4 can be achieved by B,N-doping of 50% of the pentacene units. The only way SF can take place in B,N-doped crystals is via an initial S_0_ → S_1_ excitation of the B,N-pentacene unit (2.98 eV), in order to get energies sufficiently high to proceed to ^1^T_1_T_1_ via a downhill energy pathway (by 2.01 eV). Otherwise, if the S_0_ → S_1_ photoexcitation takes place on the pentacene unit (0.72 eV, that is 2.2 eV less energy demanding in the doped crystal), SF would require going uphill in energy and would not proceed spontaneously. Figure 5 represents the energetics of the states involved in the SF process for pair1 in the pentacene crystal and pair1-ud in the doped B,N-pentacene crystal. The fact that the CT states lie in between the S_0_S_1_ and ^1^T_1_T_1_ state for the B,N-pentacene pair1-ud system implies that here the two-step mechanism is at work. This may lead to efficiency loss of the SF process as the intermediate CT state can also evolve in a separate electron–hole pair, a side process that has been reported to be important in several organic compounds,^59^ and that may partially hinder SF. In the present case, despite the sizeable coupling between the CT and ^1^T_1_T_1_ states (reflected in the large effective coupling between S_0_S_1_ and ^1^T_1_T_1_ reported in Table 3), some blocking of the SF process may occur.

#### 2.4.2 Singlet Fission from Phonon Displacements

The out-of-equilibrium geometries were taken from the selected low-energy phonon displacements listed in Table S7 for each compound. The following results would be, in part, representative of the thermal effects on the SF process. Considering a set of five phonons only for each structure limits the generality of the conclusions but helps understanding the coupling behavior of characteristic atomic movements, thus giving a detailed picture of the relevance of internal crystal distortions. Electronic couplings extracted from molecular dynamics runs, discussed in a later section, provide an alternative picture of the thermal effects.

**Figure 6:**
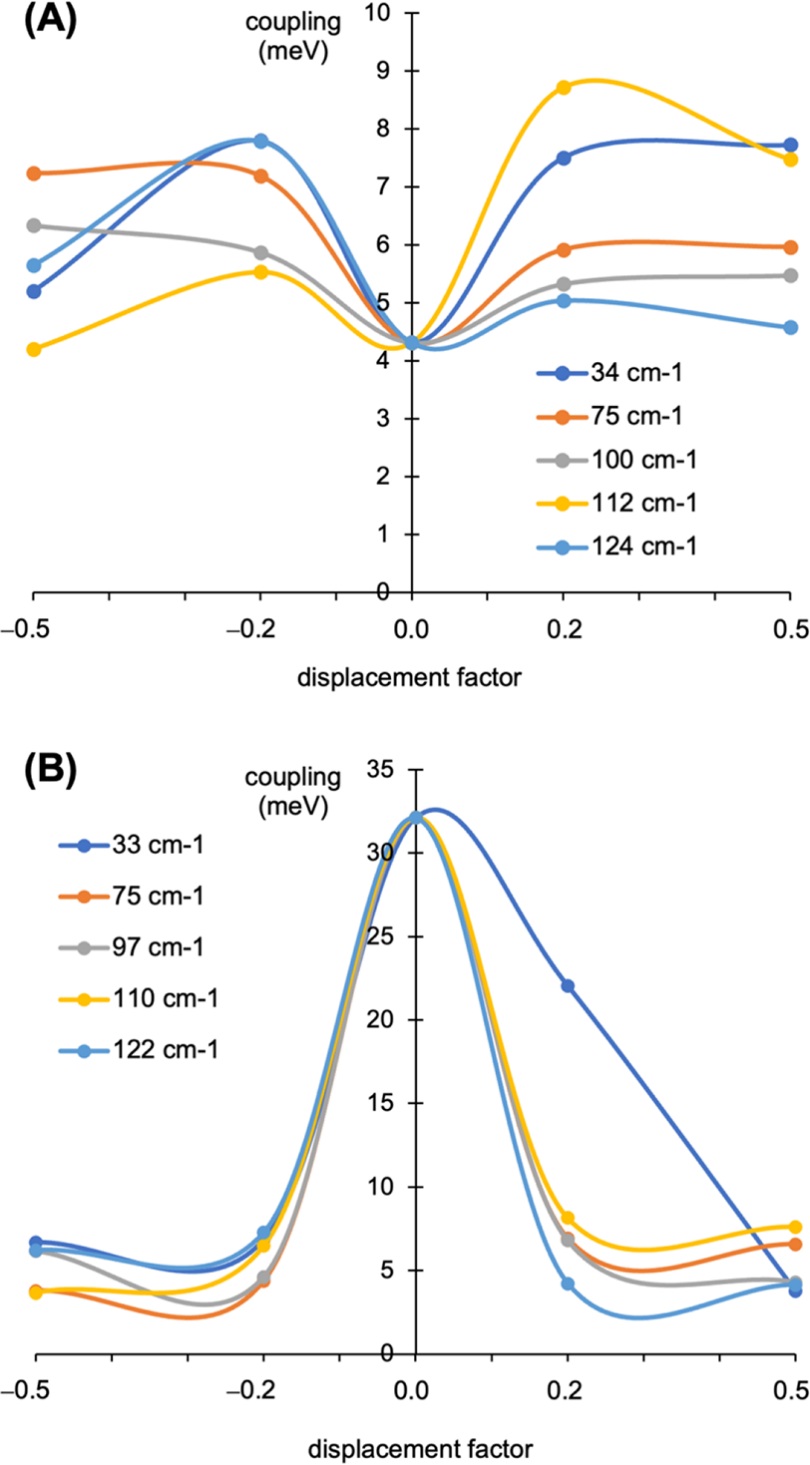
S_1_S_0_/^1^TT electronic couplings computed for selected phonons of (A) pair1 of pentacene and (B) pair1-ud of B,N-pentacene. The colored lines are guides to the eye.

The main NOCI results for pentacene and B,N-pentacene are graphically displayed in Figure 6. More numerical data are given in Table S10 in the SI. For pair1 of pentacene, the smallest atomic displacements applied (±0.2 factors) produce a notable increase of the S_1_S_0_/^1^(T_1_T_1_) coupling with respect to the reference equilibrium value of 4.32 meV. This is consistent with the observation made for the 35.0 cm^–1^ phonon by Deng et al.^41^ At larger atomic displacements (±0.5 factors), the values show different trends with respect to ±0.2 factor displacements, although not changing much. SF in pair2 (parallel intra-stack fragments) is ca. one order of magnitude weaker in comparison (0.50 meV at equilibrium geometry). Applying ±0.2 atomic displacements, we obtain notable coupling increments but still smaller than their pair1 homologues. Therefore, it can be inferred that intrastack SF is less significant in pentacene crystals if phonon displacements are considered.

The results for the B,N-pentacene crystal, shown in Figure 6B for pair1-ud and listed in Table S10 also for pair2-dd, present some differences in comparison with pure pentacene. Taking the reference of 24.7 meV for pair1-ud in its equilibrium geometry, the phonon displacements factors ±0.2 produce, in general, significantly weaker S_1_S_0_/^1^(T_1_T_1_) couplings, in general within the 4–8 meV range, thus similar to those of pentacene. One exception is the case of phonon-33 at 0.2 displacement, with a coupling value comparable to that at equilibrium (22.1 meV). Such a large value arises because of two coinciding facts: states S_1_S_0_ and D^+^D^–^ are quite close in energy at this geometry (Δ*E* = 0.011 h ≈ 300 meV) and, in addition, expressing S_1_S_0_ as
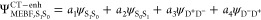
gives rather large *a*_3_ and/or *a*_4_ coefficients, indicative of an important ionic character of this wave function (see Table S11A). Also, the ^1^T_1_T_1_ MEBF presents a substantial D^+^D^–^ character as the large *b*_3_ and *b*_1_ values in the first and second columns. The combination of these results explains this large S_1_S_0_/^1^TT coupling, even if not common among the phonons studied. Small electron coupling values obtained in other cases lack one or both conditions for obtaining such large S_1_S_0_/^1^(T_1_T_1_) couplings (see Table S11B for comparison).

For the 50% doped crystal, pair2-dd results follow the trend observed for pair2 of pentacene, that is, the coupling is significantly smaller (1.16 meV) than the pair1 homologue. However, the ±0.2 phonon displacements analyzed hardly alter the equilibrium coupling (0.88 to 1.46 eV for the five phonons studied).

#### 2.4.3 Singlet Fission from Dynamical Thermal Disorder

**Figure 7:**
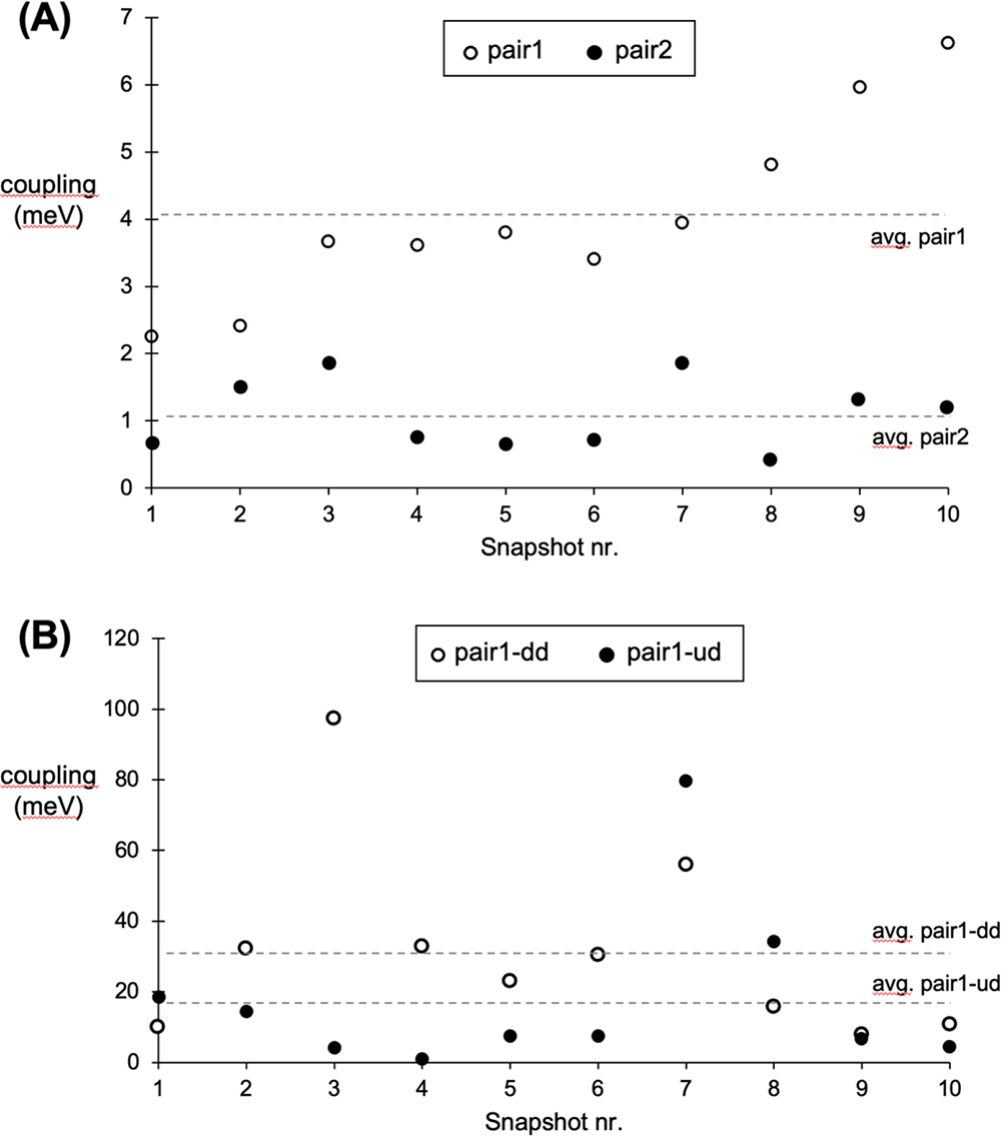
S_1_S_0_/^1^(T_1_T_1_) electronic couplings computed for snapshots ss1–ss10 for (A) pentacene, pair1 and pair2, and (B) B,N-pentacene pair1-dd and pair1-ud. Horizontal dashed lines correspond to the average values for the sets displayed. The vertical scales in panels (A) and (B) are different.

AIMD simulations explore more realistic atomic displacements than individual phonons since all atoms can move freely (within the restrictions of temperature and interatomic interactions) in any direction at any simulation step. Ten snapshot geometries (ss1–ss10) per system were used to calculate the electron couplings, as displayed in Figure 7A,B for pentacene and B,N-pentacene, respectively. For the latter, the extra-doped crystal allows us to extract the SF coupling interactions between oblique doped pentacenes (pair1-dd, Figure 3B, bottom).

The pentacene case (Figure 7A), electron couplings for pair1 (empty dots) are larger than pair2 ones (full dots). The former present couplings around an average value of 4.04 meV, very close to the value computed for the equilibrium structure (4.32 meV). Pair2 couplings (filled dots) feature all values below 2 meV around an average of 1.09 meV, larger than the coupling at equilibrium but still smaller that the pair1 couplings. In the case of B,N-pentacene, from the analysis of pair1-ud and pair1-dd, larger couplings are found (no pair2 was considered at this stage due to their expected small coupling values obtained in previous sections). From Figure 7B, most values are found below 40 meV, with only a few points in the simulation resulting in values of 80–100 meV (ss3 and ss7). Pair1-dd couplings are, on average, larger than pair1-ud couplings (24.3 vs 17.6 meV, respectively). These results suggest that one-time large distortions in the crystal might result in substantial coupling increases. The comparison of the NOCI results for ss3 (large coupling) and ss9 (small coupling) of B,N-pentacene is given in Table S12. A clear correlation exists, in each case, between the coupling strength and the contribution of the ionic configurations on the S_1_S_0_/S_0_S_1_ and T_1_T_1_ states. Thus, thermal disorder computed at 300 K seems to partially reduce the larger but to increase the smaller couplings. The results obtained with the present molecular dynamics calculations suggest that introducing the thermal effects in the crystal structure does not alter the SF phenomenon substantially. However, these effects may affect other steps of the light harvesting process, such as triplet diffusion. The study of this phenomenon is subject of ongoing research.

To rationalize the differences observed in the couplings of the characterized snapshots, we examined a set of possibly relevant structural parameters for correlations with the calculated coupling values, such as the angle between fragment planes, the loss of molecular planarity, and the distance between fragment centroids. On the one hand, for pentacene, pair1 results show some correlation in all three geometrical parameters vs the coupling values (Figure S6, left). Remarkably, we observe some increase in the coupling for large loss of planarity and also for larger interplanar angles. The distance between centroids features some increase as well, although it should be noted that the range of values covered (>0.01 Å) is most probably not significant. On the other hand, pair2 results (Figure S6, right) suggest no correlation between changes in these structural parameters and the electronic couplings obtained. Finally, the B,N-pentacene compound shows high dispersity of the results, so no correlation is inferred in this case either (Figure S7).

## 3 Conclusions

In this combined periodic DFT and ab initio computational study, we analyze the multiexciton generation process in crystals of pure pentacene and partially doped B,N-pentacene. Particularly, by the singlet fission (SF) process, a photoexcited singlet state evolves toward two singlet-coupled triplets, S_1_S_0_ → ^1^(T_1_T_1_), in which neighboring molecules (fragment *pairs*) participate, thus doubling the capacity of the material to generate electrical current.

We calculated equilibrium crystal structures for pentacene and doped B,N-pentacene. From vibrational frequency analysis (and experimental data for pentacene), we confirmed that these arrangements correspond to energy minima. Ab initio molecular dynamics provided geometries that include the thermal effects on the crystal.

The calculations involve electron correlated CASSCF wave functions and CASPT2 energies for selected fragment pairs followed by evaluation of coupling parameters based on non-orthogonal configuration interaction calculations. The B,N-doped pentacene crystal exhibits the largest S_1_S_0_/^1^TT couplings between neighboring crosswise monomers of 24.1 meV, which is ∼6 times larger than the 4.32 meV for pure pentacene. Parallel intrastack neighboring pentacene monomers present much smaller (<1 meV) S_1_S_0_/^1^TT couplings. Including phonon displacements, SF occurring via interstack crosswise neighboring pairs depends on fragment orientation. Depending on the out-of-equilibrium phonon geometries, the effects on the electronic coupling can be in opposite directions. Pentacene phonon displacements point to a general increase of the S_1_S_0_/^1^TT couplings, whereas for B,N-pentacene the homologous phonons tend to yield smaller couplings. The geometries taken from molecular dynamics simulations at room temperature give disperse coupling values with respect to equilibrium geometries. The couplings show a remarkable dependency on the inclusion of CT configurations (D^+^D^–^/D^–^D^+^) in the S_1_S_0_ ± S_0_S_1_ and ^1^(T_1_T_1_) configurations. The degree of inclusion is related to both the energy difference of the CT configurations and those that describe the lowest excited singlet and triplet states, and the interaction between the configurations. The direct interaction is relatively constant for all the geometries that were sampled in the study, with energy differences varying more strongly for the total couplings. No clear relation was found between geometrical factors and the energy difference of the CT states and the excited singlet and triplet states. Only considering equilibrium structures, following minimal energy paths connecting different stationary points, or scanning potential energy surfaces of (intra- and inter-) molecular vibrational motions appears to result in too limited an exploration of the different conformations that can be adopted due to thermal disorder. Although many of these disordered conformations do not give rise to large enhancements in the coupling, some of them lead to (nearly) degenerate CT and excited singlet and triplet states, resulting in a significant increase in the coupling between the excited singlet and the singlet coupled double triplet, which could lead to strong acceleration of the singlet fission process.

## Supporting Information Available

Optimized crystallographic parameters and stability analysis for VASP-optimized geometries of pentacene and B,N-pentacene crystals; vibrational frequencies calculated; selected phonons for singlet fission analysis; displacement matrices for phonon analysis; stability analysis on the VASP crystal geometries; comparison between different CASSCF/CASPT2 setups; excited singlet to singlet-coupled triplets couplings for phonon displacements; energies and eigenvector coefficients for the S_1_S_0_ and ^1^T_1_T_1_ MEBFs calculated for B,N-pentacene; NOCI results for snapshots of pair1-dd of B,N-pentacene. Analysis of two positional isomers of B,N-pentacene; displacement vectors of selected phonons for pentacene and B,N-pentacene; detail of the models utilized for NOCI analysis; time evolution of AIMD energies; CAS(8,8) orbitals for B,N-pentacene; S_1_S_0_/^1^T_1_T_1_ electronic couplings computed for selected phonons of pentacene; geometrical analysis of the electronic couplings obtained for AIMD snapshots (PDF)
