# Supplementary material for: Non-orthogonal Configuration Interaction Study on the Effect of Thermal Distortions on the Singlet Fission Process in Photoexcited Pure and B,N-Doped Pentacene Crystals
Source: J Phys Chem C Nanomater Interfaces. 2023 Aug 10;127(33):16249–58. doi: 10.1021/acs.jpcc.3c02083 (PMC10552079; doi:10.1021/acs.jpcc.3c02083)
Supplement: Supplementary file 1 — jp3c02083_si_001.pdf [file jp3c02083_si_001.pdf]

Supporting Information

Non-Orthogonal Configuration Interaction Study on the Effect of Thermal Distortions on the Singlet Fission Process in Photoexcited Pure and B,N-doped Pentacene Crystals

Xavier López[a], Tjerk P, Straatsma[b,c], Aitor Sánchez-Mansilla[a], Coen de Graaf[a,d]*

[a] Departament de Química Física i Inorgànica. Universitat Rovira i Virgili. Marcel·lí Domingo 1, 43007 Tarragona, Spain

[b] National Center for Computational Sciences, Oak Ridge National Laboratory, Oak Ridge, Tennessee 37831-6373, United States of America

[c] Department of Chemistry and Biochemistry, University of Alabama, Tuscaloosa, Alabama 35487-0336, United States of America

[d] Institució Catalana de Recerca i Estudis Avançats (ICREA). Passeig Lluís Companys 23, 08010 Barcelona, Spain

* Corresponding author's e-mail address: javier.lopez@urv.cat

Table S1. Optimized crystallographic parameters for the triclinic pentacene crystal, obtained as described in the main text.

Lattice parameters

7.2091936420776381	-0.0227429163603672	-2.9766315821160836
0.0043818604138568	5.8721256941819009	-1.2618176618477350
0.0294065130860065	-0.0849495519772699	15.7668236600786908

Ionic positions (fractional coordinates)

C	H	
44	28	
0.1258478485305274	0.0319468757561004	0.4124771978837203
0.8741521514694720	0.9680531242439058	0.5875228021162796
0.9737676745049531	0.7255979339375360	0.2813345041170291
0.0262323254950468	0.2744020660624625	0.7186654958829701
0.0347864305226300	0.8152637825933851	0.3754766130632238
0.9652135694773707	0.1847362174066154	0.6245233869367766
0.1530969649829921	0.1537227151079605	0.3543349497460392
0.8469030350170076	0.8462772848920395	0.6456650502539605
0.0933308448294787	0.0660628323271181	0.2556665385354676
0.9066691551705209	0.9339371676728819	0.7443334614645323
0.1218499445228336	0.1844957414500671	0.1951745573054584
0.8781500554771663	0.8155042585499332	0.8048254426945421
0.0622857259552237	0.0959681017871895	0.0978517596317079
0.9377142740447758	0.9040318982128113	0.9021482403682923
0.0905627249608482	0.2139365462691927	0.0364148694198240
0.9094372750391514	0.7860634537308078	0.9635851305801763
0.9689572301763576	0.8749745900319585	0.0601899400105103
0.0310427698236417	0.1250254099680412	0.9398100599894902
0.9411419615255043	0.7565973461802744	0.1222811268195230
0.0588580384744948	0.2434026538197266	0.8777188731804780
0.0007881471258848	0.8453628001882862	0.2181175850229015
0.9992118528741152	0.1546371998117139	0.7818824149770985
0.5500248910500183	0.5654853050768962	0.4033163935709330
0.4499751089499816	0.4345146949231036	0.5966836064290666
0.4910718686811695	0.6808535452345811	0.3312159359173490
0.5089281313188305	0.3191464547654193	0.6687840640826511
0.5091902627893850	0.5825914783093784	0.2439842258856816
0.4908097372106144	0.4174085216906211	0.7560157741143191
0.4525939953181806	0.6975904842468965	0.1700337841084147
0.5474060046818190	0.3024095157531044	0.8299662158915851
0.4744078581814839	0.6020197766562047	0.0847129957738230
0.5255921418185168	0.3979802233437961	0.9152870042261783
0.4201659247834286	0.7186781571567894	0.0104693403840511
0.5798340752165719	0.2813218428432108	0.9895306596159491
0.5565606748204570	0.3761506057705276	0.0739244631880219
0.4434393251795436	0.6238493942294723	0.9260755368119788
0.6119244686912767	0.2603236788034061	0.1491660927670765
0.3880755313087231	0.7396763211965947	0.8508339072329222
0.5910861149363222	0.3572502261507809	0.2333038963881631
0.4089138850636780	0.6427497738492197	0.7666961036118368
0.6505414643604468	0.2434314652870266	0.3106535593009224
0.3494585356395538	0.7565685347129734	0.6893464406990776
0.6308115325912450	0.3444087861323162	0.3930694557536407
0.3691884674087558	0.6555912138676837	0.6069305442463587
0.1732054746789859	0.1014190438888861	0.4875052469379438
0.8267945253210148	0.8985809561111142	0.5124947530620559
0.9037002187159368	0.5607943562366187	0.2529679352508796
0.0962997812840638	0.4392056437633817	0.7470320647491209

0.0128667906040219	0.7209047520768790	0.4224021476626273
0.9871332093959784	0.2790952479231221	0.5775978523373732
0.2207221336747178	0.3203188671327147	0.3818580542202676
0.7792778663252823	0.6796811328672862	0.6181419457797318
0.1922590410340514	0.3494072819819184	0.2234135793412491
0.8077409589659482	0.6505927180180819	0.7765864206587511
0.1605983650477402	0.3790356976007891	0.0644961531082530
0.8394016349522596	0.6209643023992115	0.9355038468917470
0.8713795162000955	0.5913697919911404	0.0942103748718448
0.1286204837999042	0.4086302080088595	0.9057896251281549
0.5355403204542147	0.6437855914245674	0.4692953815306613
0.4644596795457856	0.3562144085754329	0.5307046184693391
0.4297820852113491	0.8496969454082437	0.3393648135990192
0.5702179147886510	0.1503030545917557	0.6606351864009807
0.3911587327846319	0.8664271113058197	0.1783130481950152
0.6088412672153679	0.1335728886941803	0.8216869518049845
0.3583676654934101	0.8873233673100089	0.0186932895211680
0.6416323345065902	0.1126766326899917	0.9813067104788320
0.6740171477819074	0.0917427906672446	0.1411415289882289
0.3259828522180928	0.9082572093327559	0.8588584710117717
0.7126808225311609	0.0750268469768686	0.3027764054421772
0.2873191774688388	0.9249731530231317	0.6972235945578226
0.6769984335021363	0.2558505588906948	0.4514333726280112
0.3230015664978639	0.7441494411093056	0.5485666273719887

Table S2. Optimized crystallographic parameters for the triclinic B,N-pentacene crystal, obtained as described in the main text.

Lattice parameters

-1.5857918726452045	0.5316716645124775	7.5837835960502877
-1.5500485199714789	-5.7743386168531838	0.5707385524891392
15.5993370065768104	-1.1859825258980130	-3.1871633761810250

Ionic positions (fractional coordinates)

B	C	H	N
1	42	28	1

0.5633482863419339	0.5401022397348100	0.4048106977349003
0.1212877162230834	0.0392761779261671	0.4086349535213589
0.8652127381642106	0.9782543443358021	0.5913657938096099
0.9762450798022019	0.7298261987588356	0.2799441053383547
0.0206733702223108	0.2823694234754127	0.7205568429992943
0.0326836124773740	0.8215637461893646	0.3726209202125915
0.9571607643469282	0.1938739257510452	0.6272886458075310
0.1510938674353655	0.1597937237319420	0.3510687227877181
0.8399987702024134	0.8561030572045152	0.6496276423652855
0.0954809749753648	0.0701628163776331	0.2538830882522632
0.9036279007145650	0.9418311062238515	0.7473176636583694
0.1248216017516680	0.1881586673977713	0.1938274660906741
0.8779119844722701	0.8219864369637314	0.8078112352049355
0.0665422202339772	0.0992017873202678	0.0977111083652739
0.9400178422648018	0.9088326363344985	0.9041213866386626
0.0935685136481157	0.2176700248206923	0.0364696148005472
0.9141378012978741	0.7896272348154427	0.9654867639982911
0.9744256207305493	0.8778432445662818	0.0609832474645537
0.0329038263931479	0.1296848216769058	0.9408314521891539
0.9472892678744562	0.7591435782517503	0.1227775253922260
0.0585023888100255	0.2490496944949134	0.8788447924929257
0.0048553465752971	0.8487078949712069	0.2173105410115034
0.9964458353151031	0.1617988671044051	0.7838614516872132

0.4377828451299773	0.4343993495511969	0.5996284131154141
0.5000616429392651	0.6713432458290689	0.3286540150021371
0.4993730125089378	0.3188976441418995	0.6720508603940498
0.5155477662602479	0.5788642521394073	0.2431093510911647
0.4863725959662118	0.4184422451679420	0.7584739555047945
0.4590694474904990	0.6944592309108679	0.1680061887379967
0.5451528489867605	0.3029499710936968	0.8325435448015588
0.4798136160087678	0.6016735127403867	0.0844314852318578
0.5268358583328002	0.3987983375811306	0.9168556031517551
0.4248732415291890	0.7195674964914164	0.0098688623380739
0.5821932509606530	0.2806736130594404	0.9907072691335469
0.5607550014372449	0.3747317440222808	0.0739477426992021
0.4459862570354314	0.6256589660339787	0.9268104679822726
0.6149636787724582	0.2555788851904930	0.1478048324845252
0.3891613568287330	0.7422261650347751	0.8512725475454384
0.5947095790630382	0.3509963463376264	0.2308849530722512
0.4064200183464675	0.6450831325687538	0.7681377626430818
0.6521459826496120	0.2255705655105735	0.3033763204784108
0.3451856276724835	0.7595422602812141	0.6905249091864837
0.3607386961956790	0.6575896641177765	0.6090471480959810
0.1652086898413218	0.1100800545041753	0.4825128563741640
0.8154029559096272	0.9073855629646819	0.5171482961045681
0.9085055160497320	0.5639459427607464	0.2522377134772586
0.0907032410477705	0.4468932865330477	0.7480464148767562
0.0088661998147185	0.7284509878614103	0.4191014558403256
0.9786163864641959	0.2891404183525318	0.5807409888206873
0.2174314601172985	0.3269783380425843	0.3780142693908809
0.7698660461138456	0.6915216435304258	0.6222921702292604
0.1940299502915649	0.3536223475055545	0.2214973478149192
0.8072658490094435	0.6575178454933454	0.7802420955543019
0.1627272788033755	0.3830417459910506	0.0638420321526591
0.8446697283532169	0.6244754600827169	0.9381161501247564
0.8789454237113739	0.5933429740680340	0.0953942989670749
0.1280049604278836	0.4141636361133983	0.9062624039826642
0.5598891573673072	0.5972921082310721	0.4801847854580865
0.4488838919717074	0.3569002504971914	0.5342672619612557
0.4373231354441816	0.8401709201858465	0.3341048254946101
0.5586502194849956	0.1488447808121285	0.6649001342755633
0.3968120781254932	0.8629354214858809	0.1749015823289397
0.6050509717123619	0.1331898792532566	0.8252503834928652
0.3632648154366684	0.8883836215154970	0.0168707858685385
0.6425401813332648	0.1111825876735251	0.9833981585869863
0.6745725523584259	0.0856661507575930	0.1402414010839832
0.3279878060784571	0.9113900724904592	0.8581867913534983
0.7094502538874339	0.0547385531306690	0.2948329390159707
0.2852349940736574	0.9292843546515568	0.6975415620670367
0.6810553707520921	0.2083124248228944	0.4318263109702403
0.3157740870793279	0.7468306584415561	0.5506511636319523
0.6379301905517775	0.3124878720160278	0.3840391741869350

Table S3. Optimized crystallographic parameters for the triclinic pentacene and the B,N-pentacene crystals, obtained with two k-point meshes (2×2×2 and 4×4×4) with the VASP program. The difference in unit cell energies is shown in the rightmost column. The values correspond to application of the PBE-vdW functional.

	a (Å)	*b* (Å)	*c* (Å)	α (°)	β (°)	γ (°)	cell volume (Å^3)	*E* diff. (eV)
Pentacene								
2×2×2	7.80	6.01	15.77	102.44	112.33	85.53	667.2	0.0076
4×4×4	7.77	6.01	15.97	101.52	113.55	85.43	668.9	
B,N-pentacene								
2×2×2	7.77	6.01	15.97	101.52	113.55	85.43	668.9	0.0333
4×4×4	7.78	6.00	15.99	101.51	114.17	85.39	667.8	

Table S4. List of the 216 vibrational frequencies, in cm^{-1}, computed for the optimized crystal of pentacene. The three lowest frequencies (in italics) correspond to the acoustic modes.

3138.38	1535.71	1224.40	895.43	599.83	185.45
3138.13	1533.59	1223.14	886.89	597.59	154.85
3135.77	1532.16	1190.30	884.69	568.12	145.50
3135.44	1514.82	1188.45	876.99	566.76	140.69
3127.65	1510.95	1186.09	875.95	548.35	139.26
3127.43	1504.30	1186.03	871.73	543.36	136.32
3124.94	1502.13	1175.81	865.65	521.37	131.06
3123.82	1460.64	1174.48	849.74	515.00	128.07
3121.78	1458.83	1163.79	844.97	497.23	124.76
3121.41	1443.63	1163.53	841.51	496.18	112.20
3121.21	1441.52	1158.81	838.56	483.41	101.53
3119.20	1441.04	1156.56	837.22	481.79	100.16
3117.72	1439.67	1144.24	833.82	478.95	75.46
3116.18	1406.58	1141.14	820.33	476.62	64.42
3116.10	1404.84	1135.15	819.98	472.75	56.84
3115.93	1401.61	1129.64	789.55	470.80	53.21
3115.87	1400.88	1120.22	788.39	469.78	47.30
3114.75	1398.52	1116.53	773.62	467.75	34.44
3114.53	1397.79	1009.45	767.88	459.15	*0.116*
3113.86	1397.26	1008.88	763.93	453.14	*0.320*
3113.28	1394.82	1008.16	760.59	442.49	*0.574*
3112.67	1386.61	1005.98	754.11	441.63	
3112.01	1381.70	985.46	751.65	386.73	
3111.74	1366.17	979.78	749.66	382.32	
3110.25	1363.00	976.69	745.53	356.51	
3109.58	1341.63	971.58	743.77	354.42	
3106.80	1338.17	962.92	740.99	353.22	
3105.23	1312.94	960.18	739.56	349.79	
1621.25	1311.34	958.54	739.11	301.45	
1620.86	1289.96	957.14	737.79	296.99	
1618.53	1286.03	918.43	726.53	267.41	
1617.35	1284.50	912.97	711.43	262.25	
1594.08	1283.39	912.04	709.41	260.14	
1591.33	1269.80	911.59	699.68	255.53	
1585.49	1269.27	911.18	699.18	240.14	
1583.50	1269.17	910.46	623.07	239.24	
1547.56	1268.70	901.00	622.56	208.12	
1543.87	1234.03	899.54	618.63	194.79	
1536.51	1231.81	896.40	617.71	189.70	

Table S5. List of the 216 vibrational frequencies, in cm^{-1}, computed for the optimized crystal of B,N-pentacene. The three lowest frequencies (in italics) correspond to the acoustic modes.

3483.53	1580.94	1247.72	890.57	584.93	143.00
3143.33	1570.94	1240.61	888.22	567.10	141.73
3135.19	1541.77	1231.96	876.57	554.18	136.71
3134.53	1536.07	1222.48	875.19	545.62	131.66
3129.00	1531.93	1214.86	871.29	534.11	126.01
3126.52	1530.61	1207.06	869.79	517.92	121.76
3122.08	1525.76	1189.15	864.05	496.46	110.32
3121.87	1511.92	1185.69	852.91	494.03	102.51
3120.27	1509.01	1182.83	848.77	491.72	96.79
3119.25	1500.91	1179.02	843.46	481.81	74.99
3118.49	1497.41	1174.94	840.30	479.49	64.42
3116.65	1490.58	1168.42	838.11	478.68	57.65
3115.95	1465.37	1162.00	831.92	475.38	51.87
3114.09	1458.47	1159.69	821.05	471.13	34.23
3113.78	1443.11	1154.42	819.87	469.32	33.17
3113.43	1440.47	1142.70	819.34	467.43	*0.213*
3113.18	1439.46	1140.61	789.21	463.58	*0.291*
3112.17	1421.15	1129.87	785.28	453.80	*0.794*
3111.69	1404.67	1124.32	773.24	447.06	
3110.90	1403.51	1117.63	767.16	442.19	
3110.47	1402.10	1008.84	763.15	439.22	
3109.17	1400.28	1008.51	757.22	387.17	
3105.53	1400.10	1006.23	750.61	375.75	
3104.67	1395.87	982.17	748.64	356.98	
3099.74	1393.24	977.93	746.64	352.93	
3098.10	1382.45	971.24	744.17	351.57	
3085.51	1380.44	963.32	740.51	346.49	
2626.44	1368.64	960.99	739.59	301.19	
1621.83	1363.41	958.65	737.46	296.59	
1616.13	1341.08	957.11	732.95	267.16	
1615.12	1337.66	940.01	730.43	260.87	
1604.93	1309.99	919.48	709.89	259.34	
1589.18	1306.59	917.09	709.12	251.84	
1581.53	1289.44	915.38	702.30	239.96	
1580.94	1285.67	912.31	699.49	236.34	
1570.94	1283.72	911.48	679.92	207.82	
1541.77	1279.80	906.70	622.52	195.42	
1536.07	1274.66	905.93	621.62	190.24	
1531.93	1270.87	900.33	618.17	186.32	
1530.61	1269.71	897.65	612.41	152.53	
1525.76	1268.49	893.18	598.09	147.37	

Table S6. Frequencies and vibration modes of selected phonons for pentacene and B,N-pentacene crystals.

	Frequency[a]	Vibration mode
Pentacene	34	Lateral slide, no fragment approaching
	75	Up-down slide
	100	Slight fragment approaching
	112	Large fragment approaching
	124	Some internal deformation, loss of planarity
B,N-pentacene	33	Lateral slide, no fragment approaching
	75	Up-down slide
	97	Slight fragment approaching
	110	Large fragment approaching
	122	Some internal deformation (loss of planarity) + slight fragment approach

[a] In cm^{-1} units.

Table S7. Displacement matrices (with components d*x*, d*y*, d*z*) generated by the VASP program from the optimized pentacene and B,N-pentacene crystals. The list includes 5 phonons for each structure. Each displacement matrix was multiplied by the corresponding factor ±0.2 or ±0.5 and added to the equilibrium geometry matrix to generate a new (distorted) structure from which the fragment pair geometries for CASSCF/CASPT2 and NOCI calculations were taken.

Pentacene

Phonon 34 cm^{-1}

d*x*	d*y*	d*z*
-0.038217	0.003872	-0.132051
-0.038217	0.003872	-0.132051
-0.014074	-0.003619	-0.141416
-0.014074	-0.003619	-0.141416
-0.030006	0.000099	-0.137241
-0.030006	0.000099	-0.137241
-0.028596	0.003932	-0.130915
-0.028596	0.003932	-0.130915
-0.008276	-0.001800	-0.136172
-0.008276	-0.001800	-0.136172
0.002904	-0.005268	-0.137040
0.002904	-0.005268	-0.137040
0.018401	-0.010378	-0.140711
0.018401	-0.010378	-0.140711
0.022986	-0.012546	-0.142003
0.022986	-0.012546	-0.142003
0.021868	-0.011980	-0.142258
0.021868	-0.011980	-0.142258
0.014575	-0.010519	-0.143260
0.014575	-0.010519	-0.143260
-0.000933	-0.005399	-0.140149
-0.000933	-0.005399	-0.140149
-0.097747	-0.029390	0.145680
-0.097747	-0.029390	0.145680
-0.042075	-0.009382	0.137295
-0.042075	-0.009382	0.137295
0.012156	0.008966	0.135273
0.012156	0.008966	0.135273
0.041207	0.019003	0.131519
0.041207	0.019003	0.131519
0.064910	0.026520	0.132058
0.064910	0.026520	0.132058
0.071225	0.029237	0.132159
0.071225	0.029237	0.132159
0.067596	0.027752	0.133204
0.067596	0.027752	0.133204
0.051417	0.023368	0.136622
0.051417	0.023368	0.136622
0.018951	0.012080	0.138469
0.018951	0.012080	0.138469
-0.029378	-0.004212	0.146700
-0.029378	-0.004212	0.146700
-0.092804	-0.027152	0.150575
-0.092804	-0.027152	0.150575
-0.015519	0.001652	-0.036704
-0.015519	0.001652	-0.036704
-0.003152	-0.001557	-0.042060
-0.003152	-0.001557	-0.042060
-0.011496	0.000572	-0.039658
-0.011496	0.000572	-0.039658

-0.009529	0.001771	-0.036464
-0.009529	0.001771	-0.036464
-0.001126	-0.000601	-0.038725
-0.001126	-0.000601	-0.038725
0.006170	-0.003407	-0.040996
0.006170	-0.003407	-0.040996
0.005140	-0.003506	-0.042131
0.005140	-0.003506	-0.042131
-0.042618	-0.013667	0.042866
-0.042618	-0.013667	0.042866
-0.013726	-0.003332	0.038371
-0.013726	-0.003332	0.038371
0.009646	0.004546	0.037303
0.009646	0.004546	0.037303
0.019587	0.008049	0.038109
0.019587	0.008049	0.038109
0.014930	0.006790	0.040079
0.014930	0.006790	0.040079
-0.007034	-0.000629	0.043268
-0.007034	-0.000629	0.043268
-0.041446	-0.012770	0.045822
-0.041446	-0.012770	0.045822

Phonon 75 cm^{-1}

d*x*	d*y*	d*z*
0.005116	0.139356	-0.004736
0.005116	0.139356	-0.004736
-0.013940	0.150256	-0.005492
-0.013940	0.150256	-0.005492
-0.007057	0.146121	-0.006076
-0.007057	0.146121	-0.006076
0.010774	0.139380	-0.003804
0.010774	0.139380	-0.003804
0.008995	0.143013	-0.004461
0.008995	0.143013	-0.004461
0.008095	0.144832	-0.003649
0.008095	0.144832	-0.003649
0.007837	0.146813	-0.004413
0.007837	0.146813	-0.004413
0.001543	0.149705	-0.004026
0.001543	0.149705	-0.004026
0.003976	0.148727	-0.004622
0.003976	0.148727	-0.004622
-0.006493	0.151871	-0.004734
-0.006493	0.151871	-0.004734
-0.001482	0.147968	-0.004969
-0.001482	0.147968	-0.004969
0.013924	-0.159684	0.006808
0.013924	-0.159684	0.006808
0.012111	-0.150031	0.012384
0.012111	-0.150031	0.012384
0.003798	-0.143741	0.007363
0.003798	-0.143741	0.007363
0.002257	-0.137803	0.009994
0.002257	-0.137803	0.009994
0.007744	-0.129396	0.005437
0.007744	-0.129396	0.005437
0.005655	-0.129417	0.004694
0.005655	-0.129417	0.004694
0.008387	-0.128863	0.003068
0.008387	-0.128863	0.003068
-0.001121	-0.138265	-0.000225
-0.001121	-0.138265	-0.000225

-0.003747	-0.146749	0.001668
-0.003747	-0.146749	0.001668
-0.023672	-0.165252	-0.002261
-0.023672	-0.165252	-0.002261
-0.013297	-0.171166	0.000517
-0.013297	-0.171166	0.000517
0.002195	0.037895	-0.000846
0.002195	0.037895	-0.000846
-0.008022	0.045090	-0.001518
-0.008022	0.045090	-0.001518
-0.004492	0.043071	-0.001607
-0.004492	0.043071	-0.001607
0.004488	0.039391	-0.000918
0.004488	0.039391	-0.000918
0.001445	0.041989	-0.000319
0.001445	0.041989	-0.000319
-0.001861	0.044123	-0.000649
-0.001861	0.044123	-0.000649
-0.005344	0.045342	-0.001117
-0.005344	0.045342	-0.001117
0.008243	-0.046662	0.002832
0.008243	-0.046662	0.002832
0.005111	-0.042405	0.005669
0.005111	-0.042405	0.005669
-0.000911	-0.040313	0.004383
-0.000911	-0.040313	0.004383
-0.000745	-0.038128	0.001370
-0.000745	-0.038128	0.001370
-0.003297	-0.040811	-0.001532
-0.003297	-0.040811	-0.001532
-0.012417	-0.049764	-0.002628
-0.012417	-0.049764	-0.002628
-0.007656	-0.052531	-0.000530
-0.007656	-0.052531	-0.000530

Phonon 100 cm^{-1}

d*x*	d*y*	d*z*
0.213438	-0.086247	-0.022243
-0.213438	0.086247	0.022243
0.144283	-0.059814	-0.002386
-0.144283	0.059814	0.002386
0.194298	-0.077424	-0.012362
-0.194298	0.077424	0.012362
0.177971	-0.076385	-0.022586
-0.177971	0.076385	0.022586
0.125184	-0.057610	-0.013623
-0.125184	0.057610	0.013623
0.089541	-0.044304	-0.012597
-0.089541	0.044304	0.012597
0.043914	-0.022293	-0.006542
-0.043914	0.022293	0.006542
0.010150	-0.006482	-0.004860
-0.010150	0.006482	0.004860
0.033269	-0.016568	0.000207
-0.033269	0.016568	-0.000207
0.067102	-0.030273	0.000515
-0.067102	0.030273	-0.000515
0.111491	-0.050951	-0.005241
-0.111491	0.050951	0.005241
-0.265296	0.012924	0.007027
0.265296	-0.012924	-0.007027
-0.189155	0.018409	-0.016615
0.189155	-0.018409	0.016615

-0.117476	0.018846	-0.007672
0.117476	-0.018846	0.007672
-0.074163	0.015609	-0.023591
0.074163	-0.015609	0.023591
-0.032595	0.008461	-0.013270
0.032595	-0.008461	0.013270
-0.016386	-0.003921	-0.025454
0.016386	0.003921	0.025454
-0.016009	0.013185	0.015558
0.016009	-0.013185	-0.015558
-0.043963	0.022429	0.030744
0.043963	-0.022429	-0.030744
-0.104345	0.022032	0.022782
0.104345	-0.022032	-0.022782
-0.170800	0.020201	0.046285
0.170800	-0.020201	-0.046285
-0.259388	0.012941	0.039229
0.259388	-0.012941	-0.039229
0.071190	-0.027753	-0.008300
-0.071190	0.027753	0.008300
0.035366	-0.014425	0.002554
-0.035366	0.014425	-0.002554
0.062791	-0.022996	-0.002589
-0.062791	0.022996	0.002589
0.054349	-0.023750	-0.008321
-0.054349	0.023750	0.008321
0.027031	-0.013666	-0.004306
-0.027031	0.013666	0.004306
0.005078	-0.003070	-0.001644
-0.005078	0.003070	0.001644
0.015730	-0.006950	0.001390
-0.015730	0.006950	-0.001390
-0.091940	0.004751	-0.000415
0.091940	-0.004751	0.000415
-0.052242	0.006377	-0.011628
0.052242	-0.006377	0.011628
-0.023318	0.003898	-0.012129
0.023318	-0.003898	0.012129
-0.007923	-0.002080	-0.012028
0.007923	0.002080	0.012028
-0.008574	0.007550	0.014291
0.008574	-0.007550	-0.014291
-0.042794	0.007597	0.020227
0.042794	-0.007597	-0.020227
-0.089884	0.002245	0.015960
0.089884	-0.002245	-0.015960

Phonon 112 cm^{-1}

dx	dy	dz
-0.163958	0.016978	0.008257
-0.163958	0.016978	0.008257
-0.127554	-0.002218	0.005524
-0.127554	-0.002218	0.005524
-0.141591	0.005748	0.008442
-0.141591	0.005748	0.008442
-0.165911	0.015079	0.006145
-0.165911	0.015079	0.006145
-0.152826	0.005843	0.005075
-0.152826	0.005843	0.005075
-0.151379	0.001489	0.002000
-0.151379	0.001489	0.002000
-0.144846	-0.004808	0.002943
-0.144846	-0.004808	0.002943

-0.139791	-0.007365	0.001799
-0.139791	-0.007365	0.001799
-0.140370	-0.006973	0.003102
-0.140370	-0.006973	0.003102
-0.132469	-0.007130	0.003798
-0.132469	-0.007130	0.003798
-0.138862	-0.000955	0.004943
-0.138862	-0.000955	0.004943
0.130731	-0.022661	-0.003214
0.130731	-0.022661	-0.003214
0.109679	-0.023632	0.003781
0.109679	-0.023632	0.003781
0.131988	-0.006879	-0.000675
0.131988	-0.006879	-0.000675
0.124551	-0.005156	0.001961
0.124551	-0.005156	0.001961
0.134937	0.003704	-0.000663
0.134937	0.003704	-0.000663
0.138808	0.005927	-0.002409
0.138808	0.005927	-0.002409
0.142956	0.007483	-0.002994
0.142956	0.007483	-0.002994
0.159726	0.010634	-0.008844
0.159726	0.010634	-0.008844
0.160914	0.006595	-0.007603
0.160914	0.006595	-0.007603
0.188681	0.011782	-0.016531
0.188681	0.011782	-0.016531
0.176857	0.000040	-0.013157
0.176857	0.000040	-0.013157
-0.050018	0.007161	0.002517
-0.050018	0.007161	0.002517
-0.029327	-0.003968	0.000866
-0.029327	-0.003968	0.000866
-0.038378	0.001102	0.002614
-0.038378	0.001102	0.002614
-0.049787	0.005597	0.002337
-0.049787	0.005597	0.002337
-0.042677	0.000303	-0.000756
-0.042677	0.000303	-0.000756
-0.036701	-0.003569	-0.000293
-0.036701	-0.003569	-0.000293
-0.032682	-0.004462	0.000888
-0.032682	-0.004462	0.000888
0.030447	-0.011551	0.000348
0.030447	-0.011551	0.000348
0.020151	-0.011738	0.003758
0.020151	-0.011738	0.003758
0.029954	-0.003882	0.002080
0.029954	-0.003882	0.002080
0.037878	0.001115	-0.000686
0.037878	0.001115	-0.000686
0.047412	0.004182	-0.004009
0.047412	0.004182	-0.004009
0.058744	0.005973	-0.007096
0.058744	0.005973	-0.007096
0.054220	0.001238	-0.004729
0.054220	0.001238	-0.004729

Phonon 124 cm^{-1}

d*x*	d*y*	d*z*
0.185731	-0.075522	-0.027352
-0.185731	0.075522	0.027352
-0.079632	0.056901	0.015213
0.079632	-0.056901	-0.015213
0.048019	-0.005036	-0.004992
-0.048019	0.005036	0.004992
0.150118	-0.060339	-0.024412
-0.150118	0.060339	0.024412
0.004384	0.009377	-0.002469
-0.004384	-0.009377	0.002469
-0.021457	0.019154	-0.000823
0.021457	-0.019154	0.000823
-0.041137	0.024631	0.003629
0.041137	-0.024631	-0.003629
0.013381	-0.007144	-0.005202
-0.013381	0.007144	0.005202
-0.064301	0.036818	0.010771
0.064301	-0.036818	-0.010771
-0.120855	0.069284	0.020175
0.120855	-0.069284	-0.020175
-0.079862	0.052653	0.012955
0.079862	-0.052653	-0.012955
0.046699	0.029505	-0.013545
-0.046699	-0.029505	0.013545
-0.165092	-0.073688	0.005368
0.165092	0.073688	-0.005368
-0.156489	-0.076544	0.008538
0.156489	0.076544	-0.008538
-0.242593	-0.114011	0.017872
0.242593	0.114011	-0.017872
-0.137845	-0.065605	0.009878
0.137845	0.065605	-0.009878
-0.107469	-0.048751	0.010476
0.107469	0.048751	-0.010476
-0.021930	-0.013010	-0.000534
0.021930	0.013010	0.000534
0.016796	0.002064	-0.005416
-0.016796	-0.002064	0.005416
0.003478	-0.004255	-0.003987
-0.003478	0.004255	0.003987
0.203012	0.091815	-0.021398
-0.203012	-0.091815	0.021398
0.252882	0.123400	-0.030165
-0.252882	-0.123400	0.030165
0.087159	-0.038308	-0.012915
-0.087159	0.038308	0.012915
-0.047046	0.029015	0.009064
0.047046	-0.029015	-0.009064
0.012301	-0.000844	-0.001304
-0.012301	0.000844	0.001304
0.069959	-0.030698	-0.012989
-0.069959	0.030698	0.012989
0.004167	-0.000048	-0.002273
-0.004167	0.000048	0.002273
0.006591	-0.003706	-0.002193
-0.006591	0.003706	0.002193
-0.045614	0.025601	0.008154
0.045614	-0.025601	-0.008154
0.014690	0.010633	-0.004488
-0.014690	-0.010633	0.004488
-0.086799	-0.039318	0.004321

0.086799	0.039318	-0.004321
-0.093645	-0.043695	0.007915
0.093645	0.043695	-0.007915
-0.052106	-0.023927	0.005522
0.052106	0.023927	-0.005522
0.031401	0.012934	-0.004311
-0.031401	-0.012934	0.004311
0.092066	0.041694	-0.007910
-0.092066	-0.041694	0.007910
0.120446	0.060467	-0.012378
-0.120446	-0.060467	0.012378

B,N-pentacene

Phonon 33 cm^{-1}

dx	dy	dz
0.080530	0.172896	0.106600
-0.034343	0.121633	0.105310
-0.064951	-0.102528	-0.110003
-0.053165	0.103953	0.095769
-0.054285	-0.086357	-0.093471
-0.046844	0.117292	0.098833
-0.058713	-0.098421	-0.103110
-0.028064	0.115542	0.112764
-0.068138	-0.100411	-0.114115
-0.034699	0.098435	0.104025
-0.064982	-0.084057	-0.097113
-0.030500	0.080407	0.092706
-0.068959	-0.064782	-0.080412
-0.040582	0.047856	0.057681
-0.060910	-0.029891	-0.036981
-0.038377	0.021640	0.031125
-0.064019	-0.002323	-0.008106
-0.053828	0.036232	0.038875
-0.049011	-0.017276	-0.016207
-0.057370	0.061569	0.062468
-0.047383	-0.042903	-0.043787
-0.047992	0.090237	0.091617
-0.055581	-0.073792	-0.081147
0.013785	-0.173274	-0.173310
0.111764	0.138098	0.093628
-0.007015	-0.143446	-0.134913
0.087337	0.096663	0.083744
0.019020	-0.114309	-0.095770
0.108342	0.060840	0.049578
-0.001394	-0.084462	-0.050870
0.082146	0.021844	0.031383
0.025403	-0.051726	-0.018788
0.102713	-0.011716	-0.010983
0.004885	-0.020331	0.027640
0.031862	0.016261	0.051837
0.075495	-0.047937	-0.035617
0.010843	0.050820	0.089730
0.095566	-0.080935	-0.076558
0.036551	0.091317	0.101785
0.068935	-0.114445	-0.103093
0.012278	0.131762	0.120556
0.089335	-0.149821	-0.134866
0.063434	-0.178467	-0.165374
-0.009044	0.035843	0.027782
-0.018151	-0.027008	-0.029783
-0.018224	0.028546	0.025363
-0.014234	-0.023449	-0.024632

-0.015300	0.035154	0.025757
-0.015831	-0.029913	-0.029015
-0.006000	0.034042	0.033574
-0.021049	-0.029521	-0.033431
-0.005602	0.025031	0.029470
-0.022535	-0.020954	-0.026833
-0.007923	0.008916	0.013283
-0.021615	-0.003437	-0.006854
-0.019850	0.015381	0.014141
-0.010967	-0.009681	-0.008223
0.029116	0.064989	0.033070
-0.001850	-0.057410	-0.056852
0.043067	0.040551	0.022843
-0.012369	-0.040990	-0.038068
0.042111	0.018917	0.009183
-0.011240	-0.024738	-0.012084
0.040685	-0.002379	-0.007468
-0.009693	-0.006723	0.011838
-0.007889	0.013815	0.029643
0.038368	-0.023027	-0.024658
-0.007487	0.037483	0.037300
0.036153	-0.044111	-0.038047
0.004146	0.058490	0.035159
0.022848	-0.059384	-0.053058
0.036678	0.188657	0.130341

Phonon 75 cm^{-1}

d*x*	d*y*	d*z*
0.026454	0.159293	0.030126
-0.021592	-0.168240	-0.036344
-0.015086	-0.142256	0.012725
-0.018359	-0.151116	-0.008637
-0.018926	-0.151963	-0.005574
-0.021100	-0.162790	-0.026232
-0.019167	-0.153177	-0.005526
-0.018988	-0.160822	-0.020872
-0.013582	-0.135503	0.025882
-0.015648	-0.146680	0.004630
-0.013588	-0.135578	0.028600
-0.014206	-0.142212	0.013464
-0.012491	-0.131902	0.036140
-0.012876	-0.134711	0.028802
-0.012255	-0.130609	0.039095
-0.013172	-0.136196	0.026501
-0.012845	-0.130960	0.036578
-0.012846	-0.132529	0.032693
-0.013166	-0.134256	0.032162
-0.014762	-0.137691	0.020414
-0.015472	-0.141263	0.017758
-0.015639	-0.143468	0.009062
-0.015605	-0.141994	0.016541
0.016631	0.130807	-0.006628
0.033576	0.159036	0.014488
0.017552	0.128032	-0.007331
0.022530	0.154911	-0.009759
0.015762	0.126510	-0.005530
0.025755	0.147682	-0.014458
0.015191	0.126351	-0.004936
0.020157	0.136483	-0.005805
0.015050	0.124819	0.002620
0.022369	0.130462	-0.002223
0.012096	0.131286	-0.004917
0.012620	0.137110	-0.007759

0.019111	0.124096	0.004908
0.006488	0.152498	-0.028478
0.018857	0.125322	-0.001488
0.009586	0.161709	-0.028397
0.016799	0.127471	-0.007147
0.000775	0.182613	-0.051438
0.014640	0.136467	-0.027426
0.014721	0.137752	-0.024970
-0.006510	-0.050515	-0.016710
-0.003238	-0.037765	0.005212
-0.005122	-0.043541	-0.003088
-0.006030	-0.046092	-0.006118
-0.006642	-0.048985	-0.011479
-0.006080	-0.047330	-0.007415
-0.005812	-0.047583	-0.008297
-0.003918	-0.037253	0.010444
-0.003649	-0.042225	0.001228
-0.003276	-0.038147	0.009906
-0.003606	-0.040909	0.004245
-0.003810	-0.038348	0.009139
-0.004429	-0.040222	0.004483
-0.004712	-0.042587	0.001381
0.010992	0.047188	0.023057
0.005414	0.037319	0.000615
0.013075	0.044306	0.006472
0.005363	0.036636	-0.002173
0.008994	0.042901	-0.005746
0.003962	0.037019	-0.003530
0.006946	0.038247	-0.002851
0.002106	0.038683	-0.003979
-0.000783	0.045499	-0.012591
0.005160	0.036777	-0.002601
-0.004179	0.055962	-0.024354
0.003351	0.040735	-0.011995
0.000437	0.057755	-0.009966
0.003891	0.040894	-0.011418
0.009556	0.201884	-0.023748

Phonon 97 cm^{-1}

dx	dy	dz
0.038216	0.041259	0.175545
-0.013514	-0.041615	-0.176079
0.033361	0.148647	0.260513
-0.032751	-0.031271	-0.113484
0.022559	0.080130	0.143690
-0.027991	-0.032141	-0.154893
0.031084	0.134106	0.234523
-0.000278	-0.048754	-0.151701
0.027836	0.106752	0.190129
-0.004672	-0.048760	-0.110340
0.018158	0.055593	0.106299
0.004142	-0.050912	-0.088508
0.013373	0.023309	0.055661
0.001366	-0.046584	-0.060464
0.004684	-0.012104	0.004342
0.004513	-0.038206	-0.040350
-0.001960	-0.027174	-0.023922
-0.006466	-0.042247	-0.052223
0.007011	-0.018875	-0.006730
-0.017959	-0.039829	-0.068967
0.009389	0.004366	0.027468
-0.017336	-0.042215	-0.095731
0.017198	0.044573	0.087461

-0.037452	0.048896	-0.291759
0.053383	0.023461	0.124590
-0.036924	0.026328	-0.199020
0.027938	-0.008310	0.103596
-0.025505	0.002540	-0.113901
0.034296	-0.025199	0.077309
-0.023292	-0.015203	-0.060775
0.014855	-0.041232	0.057015
-0.015822	-0.033259	-0.006936
0.015289	-0.045516	0.044302
-0.018903	-0.035736	0.018723
-0.010490	-0.037707	0.050779
0.002917	-0.040542	0.009666
-0.021265	-0.026052	0.082445
0.000299	-0.028982	-0.024355
-0.004926	-0.013026	0.117905
-0.009591	-0.004351	-0.098306
-0.021463	0.002747	0.182210
-0.009057	0.018759	-0.182779
-0.021786	0.047733	-0.291334
-0.002886	-0.011733	-0.058923
0.011087	0.052551	0.093773
-0.014250	-0.006298	-0.026273
0.005583	0.018476	0.032826
-0.011719	-0.007321	-0.050518
0.010825	0.046470	0.082279
0.003840	-0.015055	-0.045897
0.008835	0.032661	0.057628
0.003929	-0.014275	-0.024811
0.005571	0.008664	0.018765
0.001302	-0.011211	-0.011657
-0.000370	-0.006224	-0.004033
-0.007767	-0.009643	-0.015851
0.001110	-0.000568	0.004973
0.017522	0.023239	0.057507
-0.013619	0.017399	-0.101117
0.024588	0.008567	0.028270
-0.012162	0.007060	-0.054031
0.015769	-0.007022	0.020103
-0.006975	-0.002866	-0.021042
0.006250	-0.013896	0.014288
-0.007556	-0.008939	0.001878
-0.012433	-0.007200	0.023150
0.000670	-0.009799	-0.002954
-0.016457	0.000166	0.054339
0.000331	0.003355	-0.046356
-0.003122	0.010104	0.080257
-0.006105	0.018249	-0.105469
0.001726	0.029120	0.244179

Phonon 110 cm^{-1}

dx	dy	dz
-0.029247	-0.061269	-0.123328
0.018406	-0.006201	0.130735
0.025357	0.035269	0.201855
0.025221	-0.000249	0.129459
0.027988	-0.002257	0.118396
0.018996	-0.014120	0.115693
0.027647	0.019126	0.171934
0.021172	0.011678	0.156769
0.020015	0.023482	0.168785
0.024881	0.021088	0.164715
0.020280	0.005010	0.123010

0.025505	0.025176	0.167059
0.020259	-0.000654	0.110593
0.025120	0.019504	0.150393
0.021234	0.000215	0.109256
0.023458	0.009763	0.128464
0.024723	0.005421	0.121110
0.025761	0.015796	0.143448
0.022059	-0.000076	0.107928
0.028574	0.016604	0.149029
0.022724	-0.008974	0.092442
0.026529	0.015700	0.153532
0.022613	-0.003711	0.106231
-0.038591	-0.081747	-0.132407
-0.043301	-0.052187	-0.067582
-0.053826	-0.052327	-0.118430
-0.028890	-0.013325	-0.091040
-0.036483	-0.012727	-0.139527
-0.033574	0.002960	-0.078100
-0.044905	0.010392	-0.133756
-0.023935	0.028832	-0.101823
-0.029631	0.033004	-0.134175
-0.019025	0.031866	-0.118346
-0.026989	0.037111	-0.129972
-0.019924	0.035414	-0.119140
-0.019607	0.033233	-0.136676
-0.008227	0.024800	-0.141363
-0.005835	0.017109	-0.159729
-0.014535	0.005436	-0.142032
-0.015663	-0.005823	-0.161683
0.000016	-0.003786	-0.208923
0.002095	-0.033985	-0.182520
-0.013182	-0.068944	-0.169342
0.004442	-0.004497	0.033299
0.007042	0.014314	0.071114
0.007996	-0.002571	0.031591
0.009570	-0.005275	0.024832
0.004821	-0.008378	0.026688
0.008673	0.007716	0.054064
0.004515	0.003219	0.044849
0.004148	0.009073	0.052369
0.006806	0.005962	0.045555
0.005823	-0.000126	0.031470
0.006209	0.001443	0.033958
0.007634	-0.000457	0.030579
0.008678	0.002201	0.037367
0.006683	-0.005006	0.021516
-0.011218	-0.028108	-0.033284
-0.014362	-0.033041	-0.030931
-0.019175	-0.020153	-0.004017
-0.023122	-0.017978	-0.023735
-0.013911	-0.001736	-0.014249
-0.018704	0.001617	-0.033073
-0.003860	0.008090	-0.030728
-0.008610	0.010618	-0.036171
0.002565	0.008912	-0.045131
0.004755	0.005186	-0.046848
0.007743	0.002226	-0.069504
0.009103	-0.008678	-0.055802
-0.001724	-0.008877	-0.076769
-0.000695	-0.025249	-0.050587
-0.014851	-0.033343	-0.233708

Phonon 122 cm^{-1}

dx	dy	dz
-0.023679	-0.036599	-0.102528
-0.030503	-0.126111	-0.187572
0.002647	0.049181	0.222692
0.034633	0.046975	0.080519
0.029565	-0.064634	-0.048590
0.006842	-0.050349	-0.052500
0.027556	0.001831	0.137146
-0.036783	-0.079663	-0.146635
-0.026685	0.012152	0.092524
-0.004819	0.023735	-0.008493
-0.020719	-0.044571	-0.082276
-0.009513	0.050507	0.005200
-0.035976	-0.047542	-0.137138
0.001295	0.064864	-0.001394
-0.019201	-0.021587	-0.142378
-0.001598	0.024346	-0.071132
-0.008821	0.025314	-0.068024
0.005754	0.074369	0.015140
-0.006840	-0.023152	-0.146401
0.027954	0.102786	0.098607
0.005779	-0.070403	-0.183992
0.019626	0.070922	0.075188
0.000865	-0.070147	-0.129950
-0.033766	-0.118480	0.023119
-0.035278	-0.062367	0.108101
-0.067678	-0.005585	-0.125861
-0.001137	-0.026357	0.155634
-0.030678	0.043605	-0.099655
-0.007429	-0.012363	0.222027
-0.048741	0.101772	-0.140583
0.011247	0.038247	0.170352
-0.012175	0.104660	-0.040679
0.015535	0.051774	0.119861
-0.002849	0.089939	0.024492
0.018696	0.054837	0.126450
0.009788	0.078568	0.024696
0.041258	0.025387	0.111872
0.033938	0.034917	0.029923
0.026497	0.000406	0.081251
0.015903	-0.002754	0.018595
0.041983	0.014624	-0.112511
0.053575	-0.117892	0.164588
0.024061	-0.183748	0.193038
-0.015933	-0.061052	-0.088489
-0.000056	0.029861	0.109953
0.019389	0.027344	0.047350
0.017239	-0.027371	-0.030158
0.004233	-0.020258	-0.016795
0.013252	0.007308	0.061205
-0.019185	-0.036977	-0.067738
-0.016447	0.012214	0.042897
-0.009371	0.008815	-0.007629
-0.016588	-0.009791	-0.031983
-0.000291	0.007939	-0.018482
-0.002733	0.008189	-0.017948
0.014065	0.035501	0.038534
0.008087	-0.021539	-0.055734
-0.014719	-0.016731	-0.054166
-0.016238	-0.043598	0.002447
-0.023577	-0.024673	0.050542
-0.036063	0.010952	-0.067267

-0.011626	-0.005984	0.071377
-0.026270	0.035810	-0.056769
0.007146	0.010980	0.043485
-0.003817	0.029993	-0.003310
0.020031	0.012180	0.018183
0.021867	0.002955	0.024921
0.023946	0.010842	-0.052873
0.032117	-0.044495	0.073687
0.004170	0.010642	-0.112532
0.014601	-0.077801	0.092347
0.010303	0.010852	-0.252419

Table S8. Stability analysis carried out for the VASP optimized geometries of pentacene and B,N-pentacene. Values are in GPa. Total elastic tensors were obtained from VASP frequency runs, and the eigenvalues of the stiffness matrices were obtained with the ELATE program,[a] for which all-positive eigenvalues are indicative of a stable crystal.

Pentacene

Total elastic tensor

	XX	YY	ZZ	ZX	YZ	XY
XX	14.237	11.214	6.2485	–0.53176	–0.15906	0.44514
YY	11.214	20.334	4.1601	–0.41982	0.65182	–0.64473
ZZ	6.2485	4.1601	51.233	4.3059	0.25598	0.86343
ZX	–0.53176	–0.41982	4.3059	2.4981	–0.27527	–0.47422
YZ	–0.15906	0.65182	0.25598	–0.27527	3.55200	–0.50831
XY	0.44514	–0.64473	0.86343	–0.47422	–0.50831	9.8183

Eigenvalues of the stiffness matrix

λ_1	λ_2	λ_3	λ_4	λ_5	λ_6
1.9002	3.4952	5.5306	10.002	27.012	53.733

B,N-pentacene

Total elastic tensor

	XX	YY	ZZ	ZX	YZ	XY
XX	40.809	3.3329	6.2819	–3.2882	–1.0469	–3.2614
YY	3.3329	20.787	11.072	1.2867	1.0583	0.46973
ZZ	6.2819	11.072	14.245	0.17626	–1.5126	0.13798
ZX	–3.2882	1.2867	0.17626	2.3255	1.1294	0.4158
YZ	–1.0469	1.0583	–1.5126	1.1294	8.9504	0.77975
XY	–3.2614	0.46973	0.13798	0.4158	0.77975	3.8336

Eigenvalues of the stiffness matrix

λ_1	λ_2	λ_3	λ_4	λ_5	λ_6
1.7889	3.3646	5.0291	9.8369	26.673	44.258

[a] https://progs.coudert.name/elate. R. Gaillac, P. Pullumbi, F.-X. Coudert, ELATE: an open-source online application for analysis and visualization of elastic tensors, *J. Phys. Condens. Matter* **2016**, *28*, 275201.

Table S9. CASSCF and CASPT2 energy differences (in eV) between the electronic states of pentacene involved in the SF process (S_0, S_1 and T_1), with different active spaces, from (8,8) to (14,14). These values are obtained from gas-phase DFT optimized geometries.
IMAG: imaginary shift for the zero order Hamiltonian. IPEA: standard correction introduced to the zero order Hamiltonian.

	CASSCF	CASPT2				experiment[a]
		IMAG = 0		IMAG = 0.15		
		IPEA = 0	IPEA = 0.25	IPEA = 0	IPEA = 0.25	
**CAS(8,8)**						
$\Delta E(S_1–S_0)$	3.85	1.83	2.38	1.83	2.38	1.83
$\Delta E(T_1–S_0)$	1.56	0.82	1.30	0.82	1.30	0.86
$\Delta E(S_1–T_1)$	–2.29	–1.01	–1.07	–1.01	–1.07	–0.97
**CAS(10,10)**						
$\Delta E(S_1–S_0)$	3.84	1.87	2.35	1.88	2.35	
$\Delta E(T_1–S_0)$	1.28	0.98	1.38	1.01	1.38	
$\Delta E(S_1–T_1)$	–2.56	–0.89	–0.97	–0.86	–0.97	
**CAS(12,12)**						
$\Delta E(S_1–S_0)$	3.93	1.83	2.34	1.83	2.34	
$\Delta E(T_1–S_0)$	1.78	0.64	1.24	0.68	1.25	
$\Delta E(S_1–T_1)$	–2.15	–1.19	–1.10	–1.16	–1.09	
**CAS(14,14)**						
$\Delta E(S_1–S_0)$	3.70	2.03	2.41	2.04	2.42	
$\Delta E(T_1–S_0)$	1.27	1.06	1.40	1.07	1.41	
$\Delta E(S_1–T_1)$	–2.43	–0.97	–1.01	–0.97	–1.01	

[a] M. W. B. Wilson, A. Rao, J. Clark, R. S. S. Kumar, D. Brida, G. Cerullo, R. H. Friend, Ultrafast dynamics of exciton fission in polycrystalline pentacene, *J. Am. Chem. Soc.* **2011**, *133*, 11830–11833.

The values in the table were obtained with four setups to account for the dynamical electron correlation (IMAG/IPEA combinations). Changes in the IMAG parameter result in almost negligible variations in the relative state energies, but IPEA has a notable effect. Considering the spectroscopic experimental data for pentacene (rightmost column), IPEA = 0 is the most reasonable choice and is the one used throughout the article. It is remarkable that all the relevant states are in very good agreement with the experimental data when the CAS(8,8) is used and that larger CAS do not improve the performance of our choice in the present work.

For the CAS(8,8), the mean energy gap between the active space orbitals and the immediately lower occupied orbital for pentacene is ~10 eV, and with the lowest unoccupied orbital ~1.9 eV. For B,N-pentacene, these values are very similar, though the gap between the CAS orbitals and the highest occupied is slightly more variable.

Table S10. CT-enhanced $S_1S_0/^1(T_1T_1)$ couplings for pair1 and pair2 of pentacene, and for pair1-ud and pair2-dd of B,N-pentacene with phonon-generated geometries taken from Table S7. Data in meV.

	Atomic displacement factor	Phonon (cm^{-1})				
Pentacene		**34**	**75**	**100**	**112**	**124**
pair1	–0.5	5.21	7.24	6.34	4.20	5.65
	–0.2	7.80	7.19	5.88	5.53	7.79
	equilibrium	4.32				
	0.2	7.50	5.92	5.33	8.72	5.04
	0.5	7.73	5.97	5.48	7.48	4.58
pair2	–0.2	2.01	2.98	1.63	2.99	1.87
	equilibrium	0.50				
	0.2	1.61	2.45	1.61	2.27	2.07
B,N-pentacene		**33**	**75**	**97**	**110**	**122**
pair1-ud	–0.5	6.69	3.79	6.17	3.67	6.21
	–0.2	6.85	4.38	4.63	6.49	7.28
	equilibrium	24.7				
	0.2	22.1	6.93	6.80	8.15	4.24
	0.5	3.78	6.58	4.30	7.61	4.15
pair2-dd	–0.2	0.88	1.10	1.11	1.30	1.20
	equilibrium	1.16				
	0.2	1.46	1.19	1.17	1.01	1.22

Table S11. Pair1-ud energies, in Hartree units, and eigenvector coefficients for the S_1S_0 ($a_{1\text{-}4}$) and $^1(T_1T_1)$ ($b_{1\text{-}3}$) MEBFs calculated for B,N-pentacene at A) ×0.2 and B) ×0.5 displacement factors of phonon-33, for comparison. The underlined values help explaining the trends observed.

A) Coupling value = 22.1 meV

	S_1S_0	S_0S_1	D^+D^-	D^-D^+
Energy	−1693.21097719	−1693.18966206	−1693.17828416	−1693.14964740
a_1	−0.99066019	0.05943498	0.09513794	−0.08009762
a_2	0.01710548	0.95186022	−0.28979787	0.09992171
a_3	0.06360452	0.07490448	−0.05064049	−0.99408011
a_4	0.09896874	0.27933778	0.95496787	−0.02156639

	$^1(T_1T_1)$		D^+D^-	D^-D^+
Energy	−1693.23069445		−1693.17907254	−1693.15039814
b_1	−0.99466473		0.10333583	0.01747946
b_2	0.01269496		−0.00502686	0.99991616
b_3	−0.08552455		−0.99649001	−0.00402549

B) Coupling value = 3.78 meV

	S_1S_0	S_0S_1	D^+D^-	D^-D^+
Energy	−1693.19398524	−1693.17269432	−1693.15757024	−1693.13773174
a_1	−0.99331362	0.03801013	−0.09242045	0.06016407
a_2	−0.00922864	−0.97573876	−0.20422862	0.07973149
a_3	0.08725488	0.19454510	−0.97681680	0.02538345
a_4	0.04866101	0.06291549	0.04246405	0.99601790

	$^1(T_1T_1)$		D^+D^-	D^-D^+
Energy	−1693.21314018		−1693.15849273	−1693.13784538
b_1	−0.99757061		−0.03380701	−0.06308796
b_2	−0.02763071		0.99962959	0.00442866
b_3	0.04785143		0.00571486	−0.99895212

Table S12. NOCI results for ss3 and ss9 of pair1-dd of B,N-pentacene. In the section "`new MEBFs expressed in orginal MEBFs`" it can be seen how mixed MEBFs 1-3 are for ss3 (see coefficients in bold) compared to the pure nature of the states for ss9. These results explain the different electronic couplings found.

B,N-pentacene. ss3 pair1-dd

Block 1 : Energies and eigenvectors

	−1696.51411169	−1696.50284036	−1696.48362751	−1696.43828556
S_0S_1	0.90918998	−0.32367983	−0.20886133	−0.16366879
S_1S_0	−0.24497488	−0.92427938	0.22304545	0.19417833
D^+D^-	0.14643787	0.09835691	−0.11300398	0.97918718
D^-D^+	−0.25807988	−0.15447076	−0.95240675	−0.05766322

Block 2 : Energies and eigenvectors

	−1696.54192800	−1696.48024418	−1696.44261439
T_1T_1	−0.94947791	−0.31827283	0.02632172
D^+D^-	−0.01658369	−0.01282539	−0.99980125
D^-D^+	−0.26225962	0.96675264	−0.00811430

new MEBFs expressed in orginal MEBFs

	S_0S_0	S_0S_1	S_1S_0	$^1(T_1T_1)$	D^+D^-	D^-D^+
1:	0.00000000	0.00000000	0.00000000	**−0.94947791**	**−0.01658369**	**−0.26225962**
2:	0.00000000	**0.90918998**	**−0.24497488**	0.00000000	**0.14643787**	**−0.25807988**
3:	0.00000000	**−0.32367983**	**−0.92427938**	0.00000000	0.09835691	**−0.15447076**

Electronic Couplings (meV)

	S_0S_1	$^1(T_1T_1)$	S_1S_0
S_0S_1			
T_1T_1	**−97.2088922223**		
S_1S_0	−46.2258348847	0.0000000100	

B,N-pentacene. ss9 pair1-dd

Block 1 : Energies and eigenvectors

	−1696.54328016	−1696.52621667	−1696.51335782	−1696.46700321
S_0S_1	0.99607007	0.00026047	−0.08337172	0.03208012
S_1S_0	0.01173413	−0.99530072	0.07265319	−0.06415826
D^+D^-	0.02354955	0.05241500	−0.00939007	−0.99840009
D^-D^+	0.07537264	0.07021501	0.99471031	−0.00403485

Block 2 : Energies and eigenvectors

	−1696.56758791	−1696.51317420	−1696.46730576
T_1T_1	−0.99580333	0.09198918	0.01372541
D^+D^-	0.00960631	−0.00185000	0.99996010
D^-D^+	−0.07598132	−0.99723834	−0.00113869

new MEBFs expressed in orginal MEBFs

	S_0S_0	S_0S_1	S_1S_0	$^1(T_1T_1)$	D^+D^-	D^-D^+
1:	0.00000000	0.00000000	0.00000000	**−0.99580333**	0.00960631	−0.07598132
2:	0.00000000	**0.99607007**	0.01173413	0.00000000	0.02354955	0.07537264
3:	0.00000000	0.00026047	**−0.99530072**	0.00000000	0.05241500	0.07021501

Electronic Couplings (meV)

	S_0S_1	$^1(T_1T_1)$	S_1S_0
S_0S_1			
T_1T_1	**7.8383961431**		
S_1S_0	4.7432434002	0.0000000004	

Charge-transfer enhanced coupling

The enhancement of the coupling via D^+D^- and D^-D^+ charge-transfer states is included by constructing two new MEBFs as:

$$\Psi^{CT-enh}_{MEBF,S_1S_0} = \underline{a_1\psi_{S_1S_0} + a_2\psi_{S_0S_1}} + a_3\psi_{D^+D^-} + a_4\psi_{D^-D^+}$$

$$\Psi^{CT-enh}_{MEBF,T_1T_1} = \underline{b_1\psi_{T_1T_1}} + b_2\psi_{D^+D^-} + b_3\psi_{D^-D^+}$$

The direct coupling (that is, before the 'sub-block diagonalization') is between diabatic states with no charge-transfer (CT) character at all. This leads to very small (unrealistic) couplings between S_0S_1/S_1S_0 and $^1(T_1T_1)$ pair states. To include the effect of the CT states, three new MEBFs are constructed by two different subblock diagonalizations. In the first one, we determine the eigenfunctions of the 4×4 NOCI Hamiltonian spanned by the S_0S_1, S_1S_0, D^+D^- and D^-D^+ diabatic states and, in the second one, the eigenfunctions of the 3×3 NOCI Hamiltonian spanned by $^1(T_1T_1)$, D^+D^- and D^-D^+. From the first subdiagonalization, we keep the eigenfunctions dominated by the S_0S_1/S_1S_0, configurations and, from the second subdiagonalization, we keep the eigenfunction with the largest $^1(T_1T_1)$ contribution. Then, we built a 3×3 Hamiltonian spanned by these new (CT 'contaminated') MEBFs and determine the electronic coupling between them with the equation given in Eq. 1 in the main text.

A)

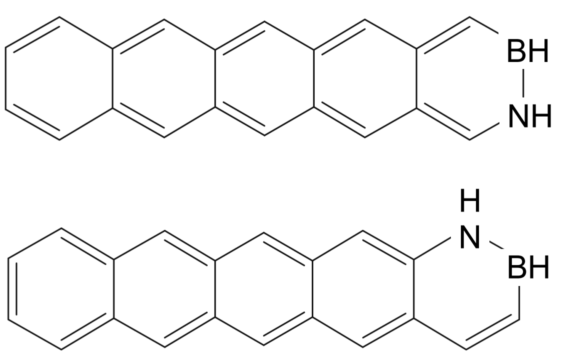

2,3 isomer (BN23)

1,2 isomer (BN12)

B)

$$\Delta E_{SF} = E(\mathrm{T}_1^{\mathrm{A}}\mathrm{T}_1^{\mathrm{B}} - \mathrm{S}_1^{\mathrm{A}}\mathrm{S}_0^{\mathrm{B}}) \quad \text{or}$$
$$= E(\mathrm{T}_1^{\mathrm{A}}\mathrm{T}_1^{\mathrm{B}} - \mathrm{S}_1^{\mathrm{B}}\mathrm{S}_0^{\mathrm{A}})$$

	doped (A)-doped (B)		doped (A)-undoped (B)			
Fragments (A, B)	A = B = BN23	A = B = BN12	A = BN23 B = pentacene		A = BN12 B = pentacene	
S_1S_0 excited state	$\mathrm{S}_1^{\mathrm{A}}\mathrm{S}_0^{\mathrm{B}} = \mathrm{S}_1^{\mathrm{B}}\mathrm{S}_0^{\mathrm{A}}$		$\mathrm{S}_1^{\mathrm{A}}\mathrm{S}_0^{\mathrm{B}}$	$\mathrm{S}_1^{\mathrm{B}}\mathrm{S}_0^{\mathrm{A}}$	$\mathrm{S}_1^{\mathrm{A}}\mathrm{S}_0^{\mathrm{B}}$	$\mathrm{S}_1^{\mathrm{B}}\mathrm{S}_0^{\mathrm{A}}$
E(CASSCF)	–0.134	0.182	0.511	–0.897	–0.169	0.099
E(CASPT2, IPEA 0.25)	0.060	0.825	0.370	–0.581	0.190	0.364
E(CASPT2, IPEA 0)	–0.304	0.583	–0.369	–0.899	–0.378	–0.002

Figure S1. A) Positional isomers 2,3 (top) and 1,2 (bottom) of B,N-pentacene. B) CASSCF and CASPT2 singlet fission energies, ΔE_{SF} in eV, for doped-doped and doped-undoped pairs of the two positional isomers represented. CASPT2 values have been obtained using with IPEA 0.25 and 0.0. The numerical values corroborate the generalized better suitability of isomer 2,3 in front of 1,2 for the singlet fission process, for which negative or close to zero ΔE_{SF} values are desired.

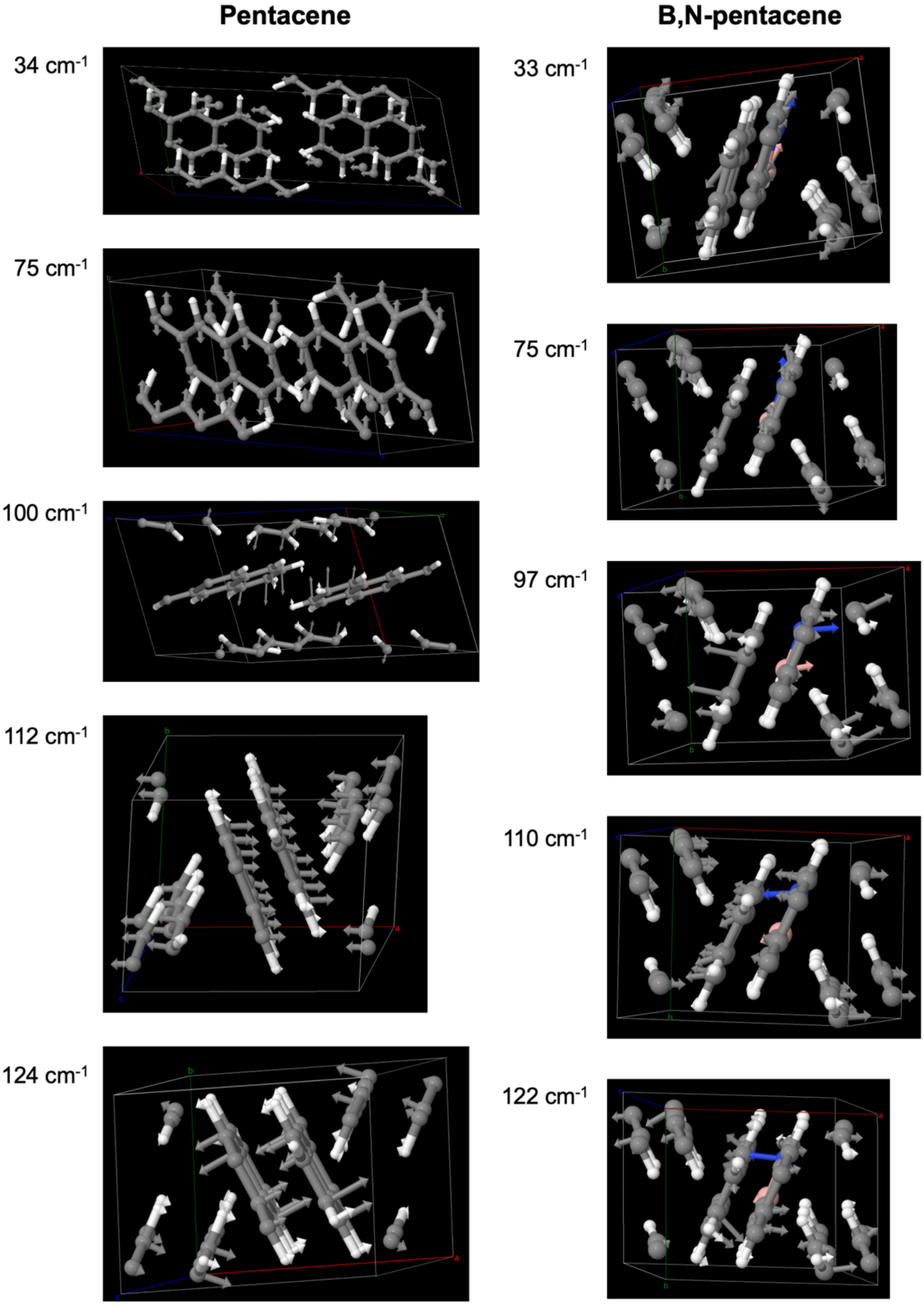

Figure S2. Displacement vectors of the selected phonons for pentacene (left) and B,N-pentacene (right).

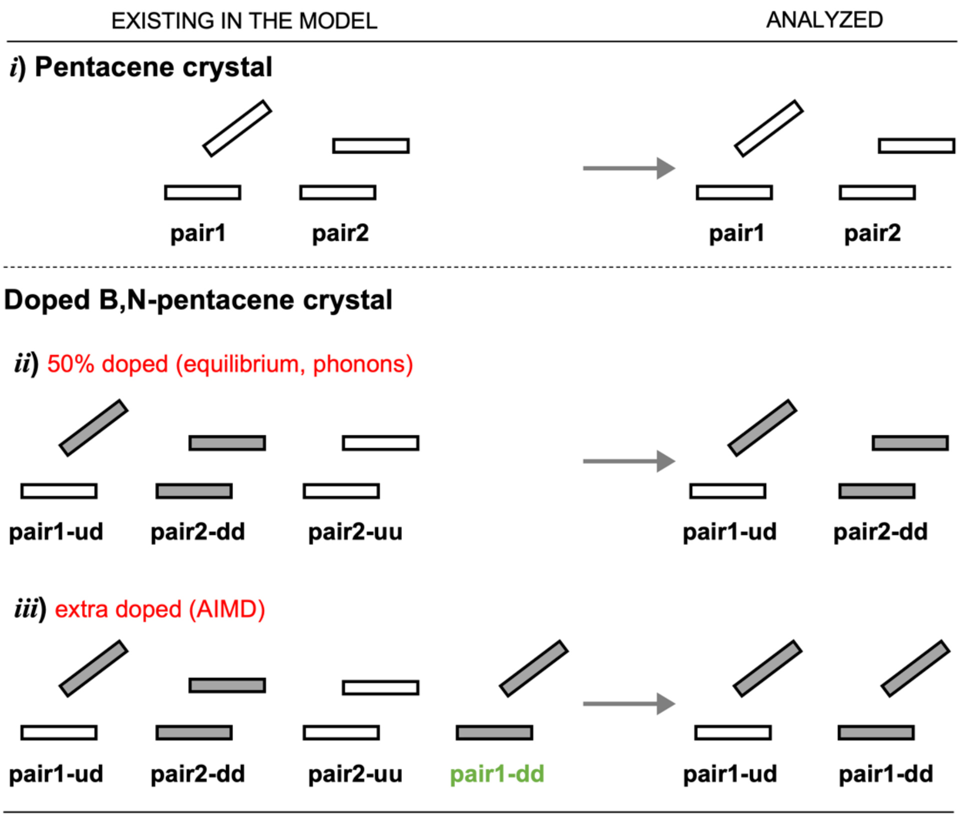

Figure S3. Scheme showing the three models utilized in this work: *i*) pentacene, *ii*) 50% doped B,N-pentacene and *iii*) extra doped B,N-pentacene crystals. Empty and filled blocks represent pentacene (u: undoped) and B,N-pentacene (d: doped), respectively. The *existing* and *analyzed* pair interactions (left and right columns, respectively) are represented in each case.

The interaction modes existing in a given B,N-doped crystal depend on the level of doping and the way the crystal is built. Taking the pentacene unit cell, with only two molecules, we only had 2 options for doping: both molecules doped or one molecule doped. Taking the latter, we obtained the 50% doped crystal. The 3-dimensional repetition of this motif generates naturally the following types of *neighboring* interactions: pair1-ud, pair2-uu and pair2-dd. At this level of doping, as the crystal model is built, pair1-dd does not exist. In the new version, we have incorporated the NOCI analysis of the latter. For the AIMD section, also a periodic calculation is carried out, but its setup is different with respect to the equilibrium geometry search. A dynamics study requires a larger simulation box (replicated in the 3 dimensions) since more degrees of freedom are allowed in the sample (periodicity repeats of the atomic motion at too short range are avoided). Assuming this, we took advantage of the larger box to introduce some extra doped molecules so that new doped-doped interactions appeared (not existing in the original 50% doped crystal). We considered this a good strategy to have access to more cases.

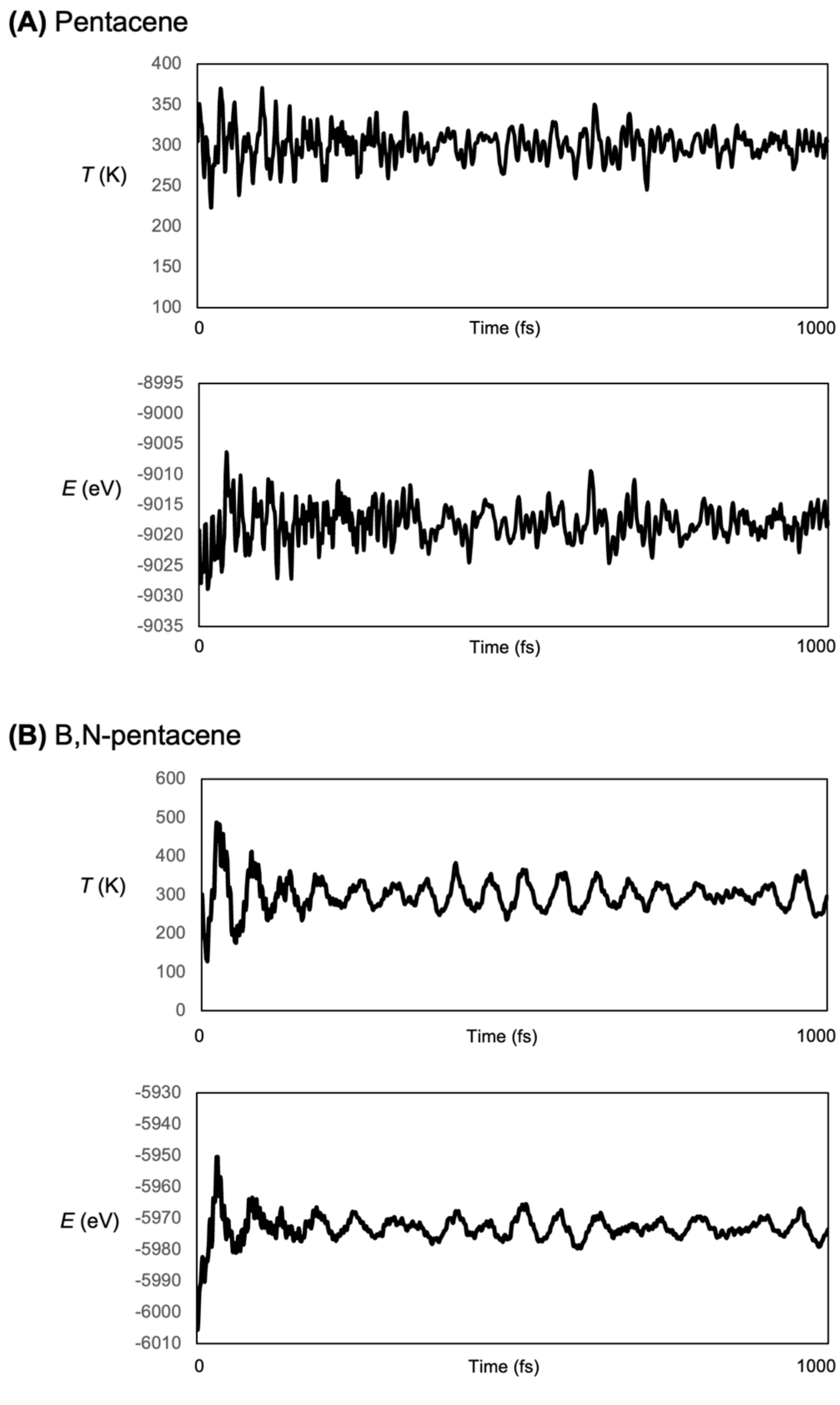

Figure S4. Ab initio molecular dynamics calculations on (A) pentacene and (B) B,N-pentacene crystals. The horizontal axis represents the time evolution.

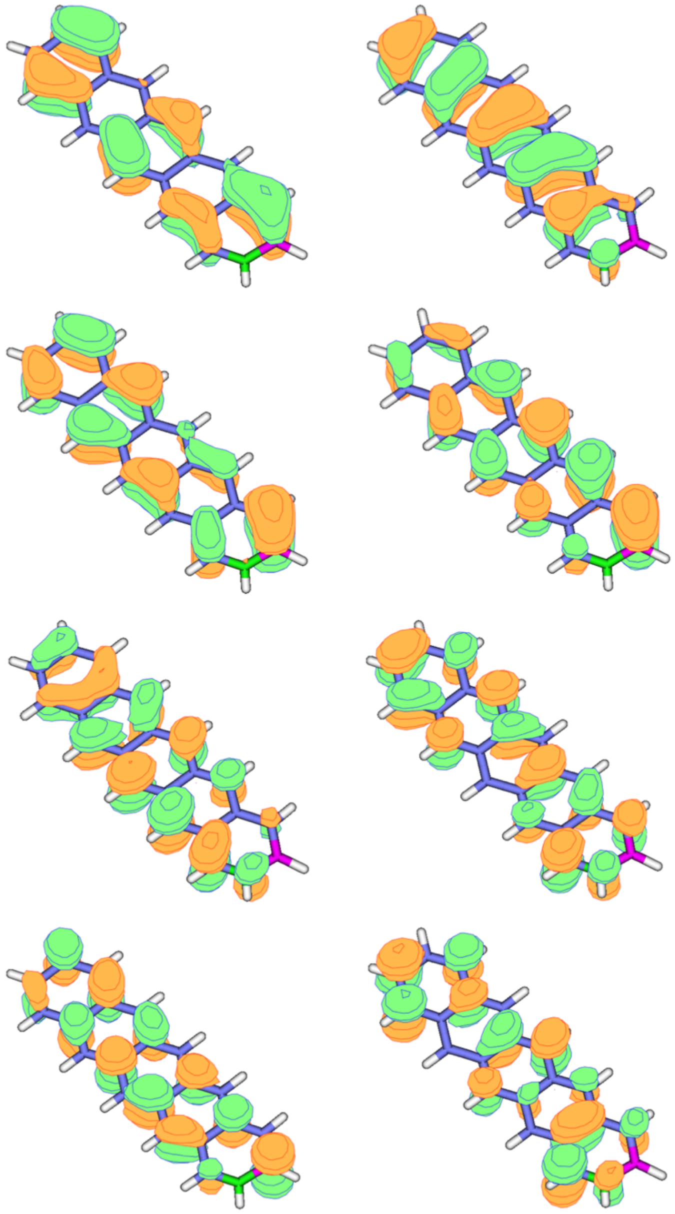

Figure S5. Orbitals of π-like nature included in the CAS(8,8) for the B,N-pentacene monomer, $BC_{20}NH_{14}$. Nitrogen and boron atoms are represented in green and magenta, respectively.

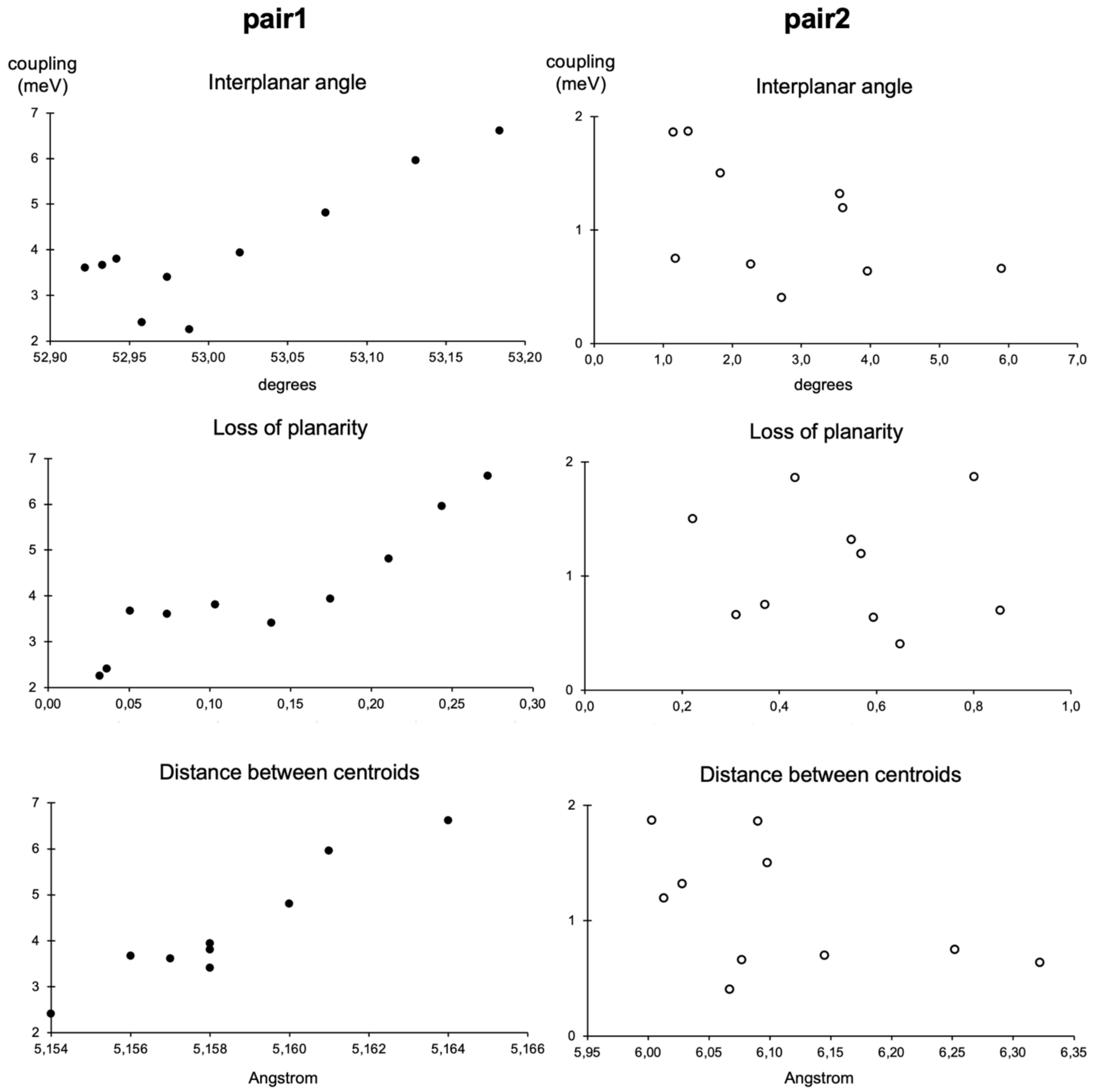

Figure S6. Graphical representation of the $S_1S_0/^1(T_1T_1)$ electronic couplings obtained for 10 snapshots taken from AIMD simulations for pentacene, as a function of the angle between fragment planes, the loss of fragment planarity and the distance between fragment centroids. Different vertical scales are used for pair1 and pair2.

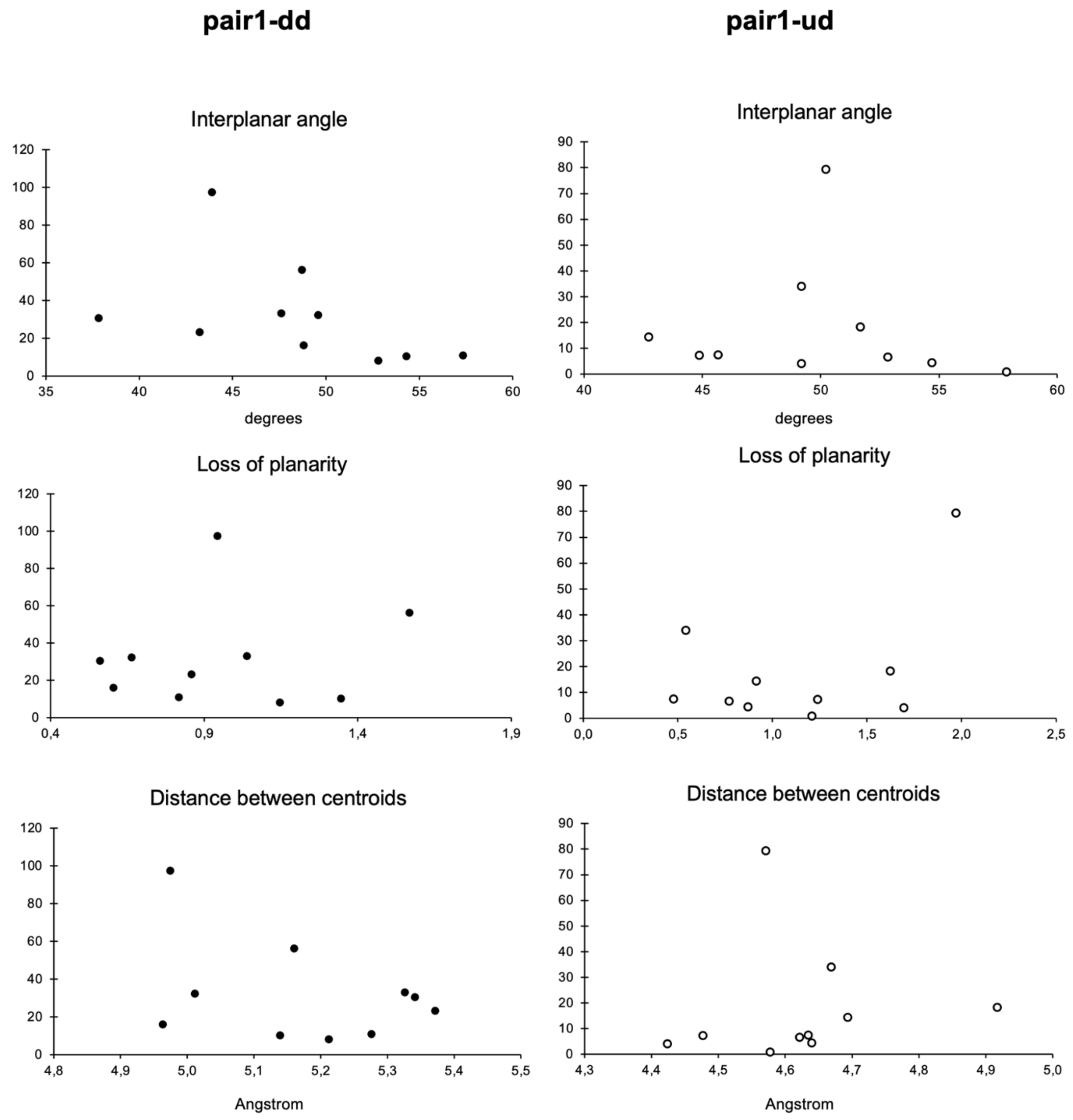

Figure S7. Graphical representation of the $S_1S_0/^1(T_1T_1)$ electronic couplings obtained for 10 snapshots taken from AIMD simulations for B,N-pentacene, as a function of the angle between fragment planes, the loss of fragment planarity and the distance between fragment centroids. Different vertical scales are used for pair1-dd and pair1-ud.
